# Spin-polarized triplet excitonic insulators in Ta_3_*X*_8_ (*X* = I or Br) monolayers

**DOI:** 10.1016/j.xinn.2026.101266

**Published:** 2026-01-16

**Authors:** Haohao Sheng, Jingyu Yao, Sheng Zhang, Quansheng Wu, Zhong Fang, Xi Dai, Hongming Weng, Zhijun Wang

**Affiliations:** 1 Beijing National Laboratory for Condensed Matter Physics, and Institute of Physics, Chinese Academy of Sciences, Beijing 100190, China; 2 University of Chinese Academy of Sciences, Beijing 100049, China; 3 Department of Physics, Hong Kong University of Science and Technology, Clear Water Bay, Hong Kong 999077, China; 4 Condensed Matter Physics Data Center, Chinese Academy of Sciences, Beijing 100190, China

**Keywords:** Bethe-Salpeter equation, excitonic insulators, spin-polarized triplet excitons, *GW* calculations

## Abstract

Bose-Einstein condensation of spin-polarized triplet excitons can give rise to an intriguing spin supercurrent, providing a direct experimental signature of exciton condensation and offering a non-dissipative channel for information transfer in spintronic devices. In this work, we predict that Ta_3_*X*_8_ (*X* = I or Br) ferromagnetic monolayers are spin-polarized triplet excitonic insulators (EIs), based on the systematic first-principles *GW* calculations coupled with the Bethe-Salpeter equation (*GW* + BSE). The single-particle calculations of spin-polarized band structures reveal that these monolayers are bipolar magnetic semiconductors, where the highest valence band and the lowest conduction band possess opposite spin polarization. The two low-energy bands, primarily originating from Ta $d_{z^{2}}$ orbitals, are almost flat. The same-orbital parity and opposite-spin nature of the band-edge states effectively suppress dielectric screening, promoting the emergence of the EI state. The *GW* + BSE calculations reveal that the binding energy of the lowest-energy exciton is 1.499 eV for the Ta_3_I_8_ monolayer and 1.986 eV for the Ta_3_Br_8_ monolayer, both of which exceed the respective *GW* band gaps, indicating spin-polarized triplet EIs. A wavefunction analysis confirms that the lowest-energy exciton is a tightly bound Frenkel-like state. Our findings establish an ideal material platform for exploring spin-polarized triplet EIs, with promising implications for spintronic applications, such as spin-current Josephson junctions.

## Public summary

• Ta_3_*X*_8_ (*X* = I or Br) monolayers exhibit a ferromagnetic insulating ground state.
• Same-orbital parity and opposite-spin nature of band edges yield low two-dimensional polarizability.
• *GW* + BSE calculations reveal a spin-polarized triplet excitonic insulator phase.
• The Bose-Einstein condensation of these excitons can give rise to an intriguing spin supercurrent.

## Graphical abstract

**Figure:**
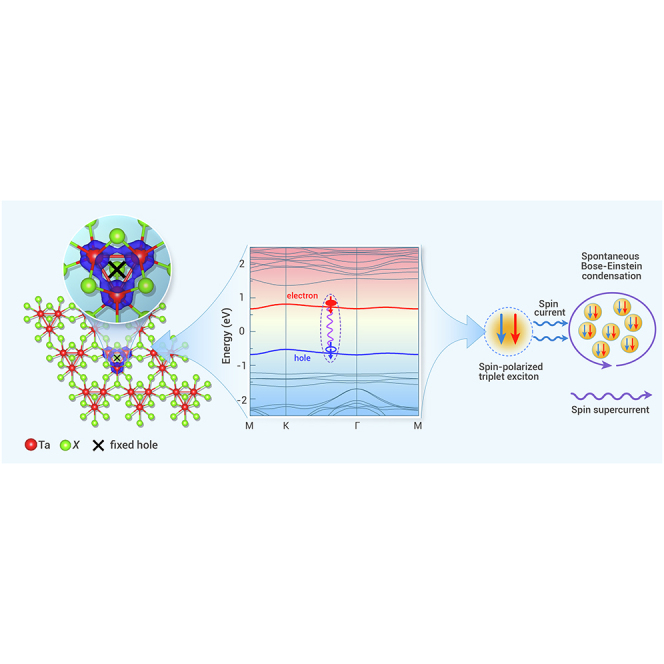

## Introduction

Excitons are electron-hole pairs bound by attractive Coulomb interactions. In semiconductors or semimetals, when the exciton binding energy (*E*_*b*_) exceeds the single-particle band gap (*E*_*g*_), the spontaneous formation of excitons can cause a renormalization of the single-particle band structure.^1^^,^^2^^,^^3^^,^^4^ This excitonic instability results in a novel many-body electronic state known as an excitonic insulator (EI). Characterized by the spontaneous Bose-Einstein condensation (BEC) of excitons,^5^^,^^6^^,^^7^ the time-reversal invariant EI behaves as a perfect insulator for both charge and spin transport. In contrast, a spin-polarized triplet EI is referred to as a spin superconductor.^8^^,^^9^ Some graphene materials in ferromagnetic (FM) phases have been theoretically predicted to host spin-polarized triplet EIs.^8^^,^^10^^,^^11^^,^^12^ Very recently, the spin-polarized triplet EI state has been experimentally observed in HfTe_5_ under magnetic fields.^13^ However, the intrinsic candidates of the spin-polarized triplet EI remain rare.

To achieve an EI, it is essential to significantly reduce the screening of Coulomb interactions. Dimensionality reduction can weaken electron-hole screening and enhance their binding in two-dimensional (2D) systems.^14^^,^^15^ Further reduction of screening can be achieved by targeting band-edge states with opposite spin components,^12^ the same parity,^16^^,^^17^^,^^18^^,^^19^^,^^20^^,^^21^ and the same *C*_2*z*_ (opposite *M*_*z*_) symmetry eigenvalues.^22^ To date, the experimentally confirmed 2D EIs have been limited to the InAs/GaSb quantum well^23^ and the monolayer of 1*T*′-phase WTe_2_.^24^^,^^25^ On the other hand, the 2D Kagome lattice offers a valuable platform for exploring the interactions between geometry, topology, correlation, multiferroicity, and more.^26^^,^^27^^,^^28^^,^^29^^,^^30^^,^^31^ In particular, niobium halide clusters are noteworthy due to their breathing Kagome geometry,^32^ which supports flat bands.^33^^,^^34^^,^^35^^,^^36^^,^^37^^,^^38^ This material family features weak interlayer van der Waals interactions, allowing for straightforward thinning down to a 2D limit through mechanical exfoliation.^39^^,^^40^ Recently, Ta_3_*X*_8_ (*X* = I or Br) monolayers have been predicted to be stable 2D intrinsic multiferroic semiconductors with the coexistence of FM, ferroelectric, and ferrovalley orders.^41^^,^^42^^,^^43^ However, their exciton properties are still unrevealed.

In this work, we predict that Ta_3_*X*_8_ monolayers are spin-polarized triplet EIs, as demonstrated by systematic first-principles *GW* calculations coupled with the Bethe-Salpeter equation (*GW* + BSE). The single-particle calculations reveal that these monolayers are bipolar magnetic semiconductors (BMSs), where the highest valence band (VB) and the lowest conduction band (CB) are fully spin-polarized in opposite spin directions. Moreover, the two low-energy bands from the Ta $d_{z^{2}}$ orbitals exhibit minimal energy dispersion, forming flat bands. The characteristics of same-orbital parity and opposite-spin band-edge states effectively suppress band-edge transitions and dielectric screening, facilitating the realization of the EI state. The *GW* + BSE calculations show that the *GW* band gap is 1.331 (1.722) eV, while the *E*_*b*_ of the lowest-energy exciton reaches 1.499 (1.986) eV for the Ta_3_I_8_ (Ta_3_Br_8_) monolayer, exceeding the corresponding *GW* band gap. A wavefunction analysis confirms that the lowest-energy exciton is a spin-polarized triplet state. The results indicate that FM Ta_3_*X*_8_ monolayers are spin-polarized triplet EIs, where spontaneous exciton BEC can generate an intriguing spin supercurrent.

## Materials and methods

We carried out first-principles calculations based on density functional theory with the projector augmented wave method,^44^^,^^45^ as implemented in the Vienna *Ab initio* Simulation Package.^46^^,^^47^ The generalized gradient approximation in the form of the Perdew-Burke-Ernzerhof (PBE) functional^48^ was used for the exchange-correlation potential. Phonon spectra were obtained using the finite-difference method, as implemented in the Phonopy package.^49^ The full-frequency single-shot *GW* calculations (*G*_0_*W*_0_)^50^^,^^51^^,^^52^^,^^53^ at the PBE level were performed to obtain a more accurate band structure. In order to analyze excitonic properties, we solved the BSE^54^^,^^55^ on top of the *GW* electronic structure (*GW* + BSE). More details of the calculation methods are provided in section A of the supplemental materials and methods.

## Results and discussion

### Crystal and electronic structures

**Figure 1:**
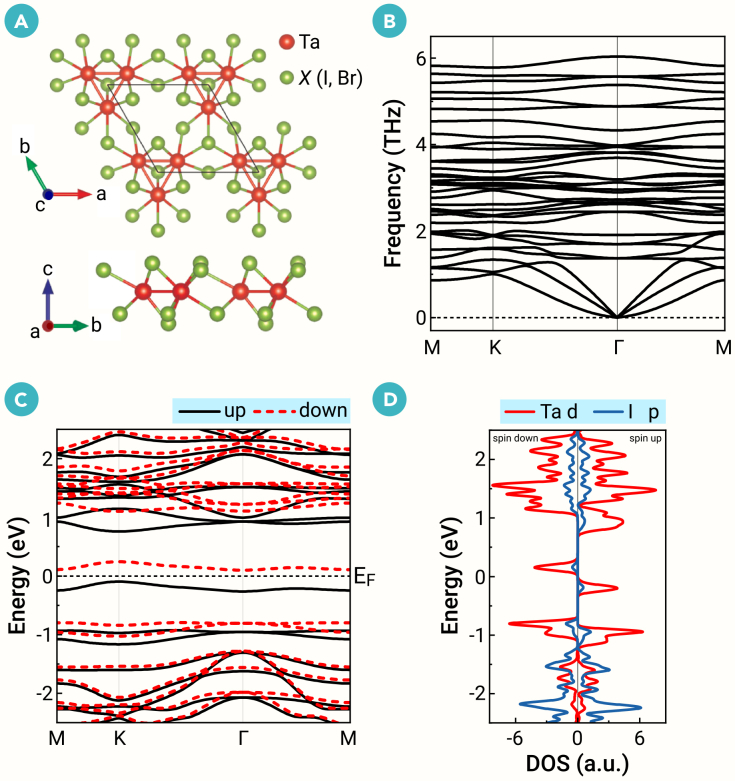
Crystal and electronic structures The (A) crystal structure, (B) phonon spectra, and (C and D) electronic band structures of Ta_3_I_8_ monolayer. The unit cell is outlined in black in (A). (C) The spin-polarized band structure and (D) partial densities of states (DOSs) without spin-orbit coupling (SOC). Black and red dotted lines represent the spin-up and spin-down bands, respectively.

**Figure 2:**
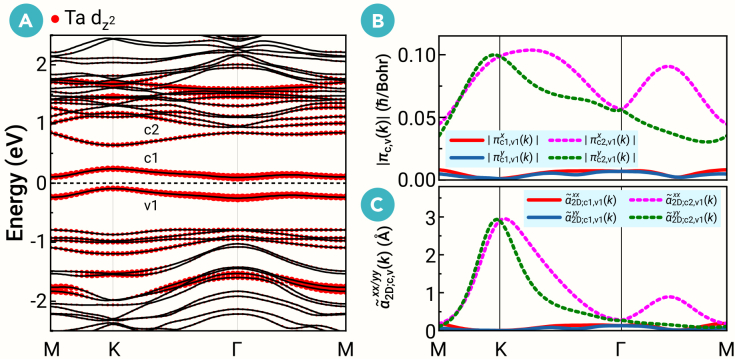
Fatband structure and 2D polarizability (A) The orbital-resolved band structure with SOC of Ta_3_I_8_ monolayer. The size of the red dots represents the weight of the Ta $d_{z^{2}}$ orbitals. (B) The modulus of the generalized momentum matrix elements ***π***_c1(c2),v1_(***k***) along the high-symmetry paths. (C) 2D polarizability ${\tilde{\alpha }}_{2D;c1\left(c2\right),v1}^{xx/yy}\left(k\right)$ along the high-symmetry paths.

The Ta_3_*X*_8_ monolayers form a breathing Kagome lattice with space group *P*3*m*1, as illustrated in Figure 1A. The structure is derived by removing a Ta atom from the 2 × 2 supercell of the 1*T*-phase Ta*X*_2_ and introducing a breathing distortion, resulting in Ta-trimer clusters in the Kagome lattice. First-principles single-particle calculations show that they exhibit an FM ground state with a total magnetic moment of 1 *μ*_*B*_ per unit cell. Since Ta_3_*X*_8_ monolayers have the same properties, we take the Ta_3_I_8_ monolayer as an example in the main text (see the results for the Ta_3_Br_8_ monolayer in section F of the supplemental materials and methods). The phonon dispersion of the FM state of the Ta_3_I_8_ monolayer is presented in Figure 1B, indicating that it is dynamically stable. Detailed analysis of structural stability can be found in section B of the supplemental materials and methods. In the spin-polarized band structure shown in Figure 1C, the highest VB and the lowest CB exhibit opposite spin directions, a hallmark feature of BMSs.^56^^,^^57^^,^^58^ Moreover, the two low-energy bands are almost flat (with ∼0.1 eV bandwidths), suggesting strong interelectronic correlations within the system. Considering spin-orbit coupling (SOC), the band structure is shown in Figure 2A, demonstrating that it has little impact on the two flat bands. From the partial densities of states in Figure 1D and the orbital-resolved band structure in Figure 2A, we find that the low-energy flat bands are mainly from Ta $d_{z^{2}}$ orbitals.

### Forbidden band-edge transition

In 2D materials, a previous study has shown that *E*_*b*_ often scales with *E*_*g*_, typically as *E*_*b*_ ≈ *E*_*g*_/4.^59^ Therefore, to achieve a 2D EI, one has to break this relationship in the materials by reducing the dielectric constant (screening of Coulomb interactions). For this purpose, we directly compute the 2D polarizability *α*_2*D*_ (2D static dielectric constant)^59^^,^^60^ in the random phase approximation approach and in the absence of the local field effect,^61^

(Equation 1) $$\alpha_{2D}^{\beta \beta }=L\frac{\varepsilon^{\beta \beta }-1}{4\pi }=\frac{1}{N_{k}}\sum_{c,v,k}{\tilde{\alpha }}_{2D;cv}^{\beta \beta }(k)\text{,}{\tilde{\alpha }}_{2D;cv}^{\beta \beta }\left(k\right)=\frac{1}{4\pi S}\frac{8\pi e^{2}\hbar^{2}}{m_{e}^{2}}\frac{\pi_{cv}^{\beta }\left(k\right)\pi_{vc}^{\beta }\left(k\right)}{{\left(E_{c,k}-E_{v,k}\right)}^{3}}\text{,}$$

where *L* denotes the vacuum thickness in the *z* direction, *S* is the area of the unit cell in the *xy* plane, *ε*^*ββ*^ denotes the static dielectric constant of the 3D system, *N*_***k***_ is the number of ***k*** points, and ***π***_*cv*_(***k***) represents the generalized momentum matrix including SOC, given by $\pi_{cv}\left(k\right)\equiv \left\langle u_{c,k}\left|\hat{p}+\frac{1}{2mc^{2}}\left(\hat{s}\times \nabla V\left(r\right)\right)\right|u_{v,k}\right\rangle$. Here, $\hat{p}$ is the momentum operator, *V*(***r***) is the potential in the crystal, $\hat{s}$ is the spin momentum operator, and *u*_*c*,***k***_ and *u*_*v*,***k***_ refer to the periodic parts of CB and VB Bloch states, respectively. Calculated by the VASP2KP,^62^ the obtained ***π***_c1(c2),v1_(***k***) and ${\tilde{\alpha }}_{2D;c1\left(c2\right),v1}^{xx/yy}\left(k\right)$ along the high-symmetry paths are presented in Figures 2B and 2C, where v1 and c1(c2) are the highest VB and the lowest (second-lowest) CB, respectively. One can see that ***π***_c1,v1_(***k***) is extremely low, less than 0.01 ℏ/Bohr. This result implies that optical transitions between the v1 and c1 bands are essentially forbidden; therefore, these bands make a negligible contribution to dielectric screening, with ${\tilde{\alpha }}_{2D;c1,v1}$ only 0.07 Å. As a result, the total 2D polarizability (*α*_2*D*_ = 5.20 Å) is very low, much smaller than that of conventional 2D materials with similar band gaps (*α*_2*D*_ ∼ 30 Å).^59^ These results clearly indicate a significant suppression of dielectric screening due to the low-energy v1 and c1 bands, which can decouple *E*_*b*_ from *E*_*g*_ and significantly enhance *E*_*b*_, enabling the realization of the EI state in the monolayer. We attribute the minor value of ${\tilde{\alpha }}_{2D;c1,v1}$ to the following reasons. First, the low-energy flat Ta *d* orbital bands suggest that Ta atoms maintain good localized atomic characteristics. According to the selection rules for atomic orbital transitions, the low-energy *d*-*d* transitions are parity forbidden. Second, in systems with weak SOC, electric-dipole transitions adhere to the spin selection rule.

### Excitons with direct transition

**Figure 3:**
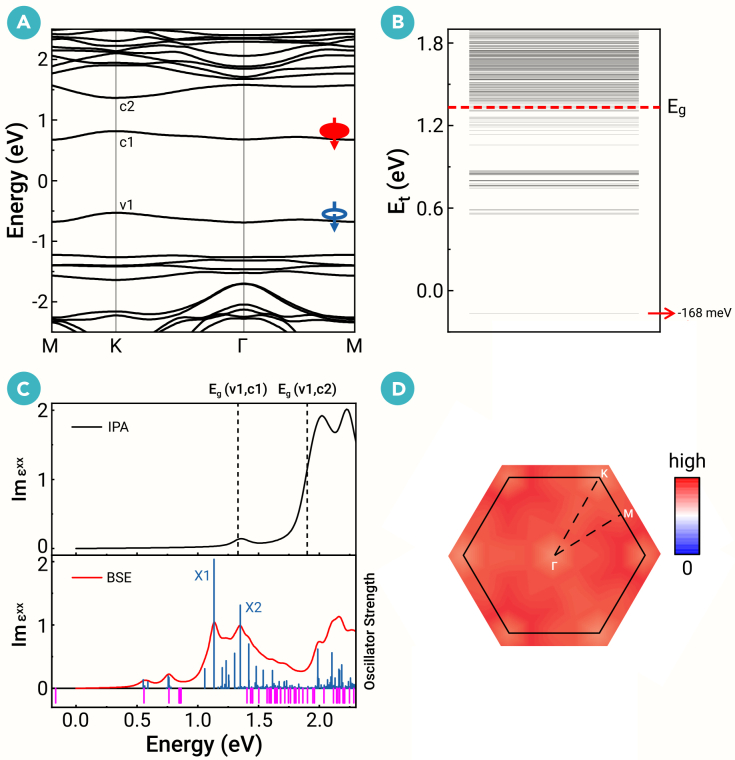
Low-energy direct excitons of Ta_3_I_8_ monolayer (A) The *G*_0_*W*_0_ band structure with SOC. The lowest-energy exciton generated by the transition between the v1 and c1 bands is shown. The blue arrow with a circle denotes the spin of the hole left behind after excitation, and the red arrow denotes the spin of the electron. (B) Exciton transition energy (*E*_*t*_) spectrum. Each horizontal line corresponds to an exciton state. The lowest-energy exciton exhibits a negative *E*_*t*_. (C) (Top) Imaginary part of the dielectric function under the independent-particle approximation (IPA), i.e., ignoring electron-hole interactions. (Bottom) Imaginary part of the dielectric function (left axis) and exciton oscillator strength (right axis) from the BSE calculation. The bright excitons are depicted by blue vertical lines, with the height indicating the oscillator strength, while the dark excitons are shown by the pink vertical lines under the *x* axis, whose strengths are less than 5 × 10^−6^ that of the brightest exciton. (D) Exciton wavefunction in reciprocal space for the lowest-energy exciton. Its substantial delocalization in reciprocal space corresponds to a high localization in real space.

To analyze low-energy excitons, we perform *GW* + BSE calculations at the PBE level (section C of the supplemental materials and methods). As shown in Figure 3A, a more accurate band structure is obtained by many-body *G*_0_*W*_0_ calculations, where the *E*_*g*_ changes from 0.334 (PBE) to 1.331 (*G*_0_*W*_0_) eV. Based on the *G*_0_*W*_0_ electronic structure, we solve the BSE using ten VBs and ten CBs. Figure 3B shows the exciton transition energies (*E*_*t*_) for all direct excitons. The lowest-energy exciton exhibits a negative *E*_*t*_ = −168 meV, indicating that the *E*_*b*_ exceeds the *E*_*g*_. This implies that the Ta_3_I_8_ monolayer exhibits a many-body EI ground state. This result is also checked by the *GW* + BSE calculations at the PBE+U and HSE06 levels (section D of the supplemental materials and methods). The lowest-energy exciton is identified as a dark state due to its extremely low oscillator strength. The imaginary part of the frequency-dependent dielectric function under the independent-particle approximation (IPA) is shown in the top image of Figure 3C. We can see very weak optical absorption at the band edge (*E*_*g*_(v1,c1)) because the band-edge transition is effectively suppressed, which is consistent with the low ${\tilde{\alpha }}_{2D;c1,v1}$. The imaginary part of the frequency-dependent dielectric function from the BSE calculations is presented in the bottom image of Figure 3C. Compared to the IPA, two prominent optical absorption peaks emerge, originating from two bright excitons (X1 and X2). These exciton wavefunctions show that X1 and X2 excitons arise from optically allowed transitions between the v1 and c2 bands.

**Figure 4:**
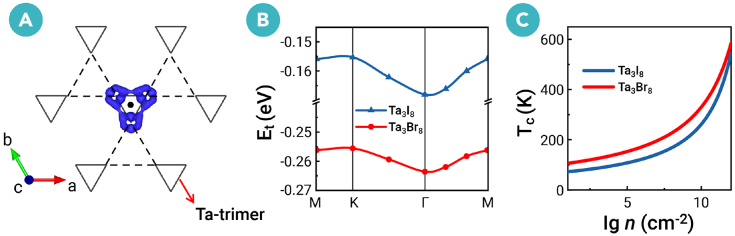
Exciton real-space wave function, dispersion, and critical temperature (A) Exciton wavefunction in real space of the lowest-energy exciton for Ta_3_I_8_ monolayer. Only the Ta-breathing Kagome lattice and a portion of the 6 × 6 × 1 supercell are shown. The contour plot (blue) is the probability density of locating the bound electron once the hole position is fixed (black dot). (B) Exciton dispersion of the lowest-energy exciton along the high-symmetry paths for Ta_3_*X*_8_ monolayers. (C) The critical temperature (*T*_*c*_) of exciton condensation as a function of the exciton density (lg*n*) for Ta_3_*X*_8_ monolayers.

We primarily focus on the lowest-energy exciton. Figure 3D illustrates its reciprocal-space wavefunction, which extends across the entire Brillouin zone (BZ). Its substantial delocalization in reciprocal space corresponds to a high localization in real space, resembling a tightly bound Frenkel-like exciton. As shown in Figure 4A, when a hole is introduced at the center of the Ta trimer, the bound electron becomes localized exclusively on the nearest Ta atoms. We further find that the lowest-energy exciton arises solely from the v1 and c1 bands. The spin-flipping in spin-polarized electron-hole transitions can produce spin-polarized triplet excitons with a finite spin moment (*s*_*z*_ = ℏ). This spin-polarized triplet EI state remains robust under weak external fields (section E of the supplemental materials and methods).

### Exciton dispersion

The **q**-dependent lowest *E*_*t*_ of Ta_3_*X*_8_ monolayers along the high-symmetry paths is shown in Figure 4B. The *E*_*t*_ remains negative across all **q** values, which indicates that the EI state is accessible for all momenta in the full BZ. Although Ta_3_*X*_8_ monolayers host an indirect *E*_*g*_, the direct exciton with **q** = 0 is energetically more stable than those with **q** ≠ 0. This indirect-to-direct transition crossover between the single-particle band dispersion and the exciton dispersion is related to nonlocal dielectric screening.^12^ In addition, Ta_3_*X*_8_ monolayers exhibit small exciton dispersion with bandwidths narrower than 13 meV, a direct consequence of their electronic flat bands. The momentum-dependent dispersion of excitons may be experimentally probed using momentum-resolved electron energy loss spectroscopy or resonant inelastic X-ray spectroscopy.^63^^,^^64^^,^^65^^,^^66^^,^^67^

### Critical temperature

Unlike parabolic bands, the low-energy flat bands can make excitons condense into an ideal form, such as one-body bosons.^68^ For spontaneously formed excitons treated as an ideal Bose gas,^18^^,^^69^^,^^70^^,^^71^ the condensation critical temperature (*T*_*c*_) as a function of the exciton density (*n*) is given by

(Equation 2) $$n=-\frac{mk_{B}T_{c}}{2\pi \hbar^{2}}\ln\left(1-e^{-\left|E_{t}\right|/\left(k_{B}T_{c}\right)}\right)\text{,}$$

where exciton mass *m* = *m*_*e*_ + *m*_*h*_ is obtained by fitting the band dispersion. Experimentally, spontaneous exciton condensation has been realized at a density of up to 10^11^–10^12^ cm ^−2^ in the 1*T*′-WTe_2_ monolayer.^24^^,^^25^ As presented in Figure 4C, with a density *n* = 10^11^ cm^−2^, the *T*_*c*_ values of Ta_3_I_8_ and Ta_3_Br_8_ EIs are 355 and 420 K, respectively. These results imply that the EI state may remain stable at room temperature. This contrasts sharply with typical Bardeen-Cooper-Schrieffer (BCS) superconductors, whose transition temperatures are usually only 1–10 K. The reason is that strong electron-hole pairing in EI is driven by direct Coulomb attraction, while weak electron pairing in BCS superconductors is mediated by phonons.

## Conclusion

In summary, we predict that Ta_3_*X*_8_ FM monolayers are spin-polarized triplet EIs, based on systematic first-principles *GW* + BSE calculations. Single-particle calculations reveal that these monolayers are intrinsic BMSs. The calculated *E*_*b*_ of the lowest-energy exciton exceeds the *E*_*g*_, indicating that Ta_3_*X*_8_ monolayers belong to a spin-polarized triplet EI ground state. Analysis of the exciton wavefunction further reveals that the lowest-energy exciton is a tightly bound Frenkel-like state. The estimated *T*_*c*_ of exciton condensation could reach room temperature at a proper exciton density. This study offers an ideal material platform for developing next-generation spintronic devices with spin-manipulation capabilities.

**Figure 5:**
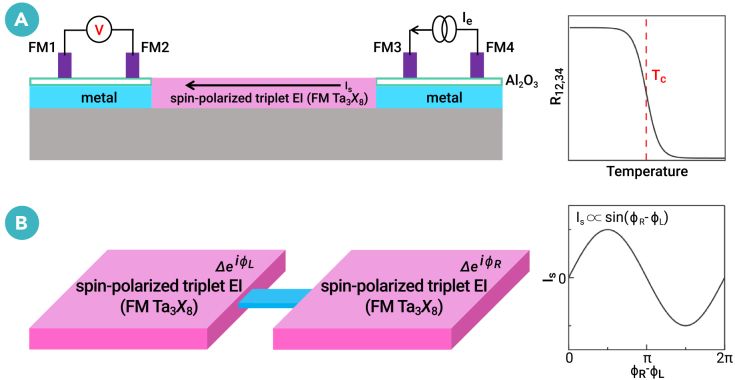
Four-terminal device and spin-current Josephson junction (A) Schematic diagram of the four-terminal device to detect spin supercurrent as a direct experimental signature of spin-polarized triplet exciton condensation. The measurable nonlocal resistance *R*_12,34_ as a function of temperature is shown. (B) Schematic diagram of the spin-current Josephson junction with a spin-superconductor/barrier/spin-superconductor geometry. At equilibrium, the spin supercurrent *I*_*s*_ as a function of the phase difference between the right and left spin superconductors (*ϕ*_*R*_ − *ϕ*_*L*_) is shown.

Unlike the time-reversal invariant EIs,^23^^,^^24^^,^^25^ which are perfect insulators for both charge and spin transport, the spin-polarized triplet EIs in FM Ta_3_*X*_8_ monolayers have a finite spin moment, giving rise to an intriguing spin supercurrent. On the one hand, we suggest a four-terminal device^8^ in Figure 5A to detect the spin supercurrent as a direct experimental signature of this exciton condensation. After a current *I*_34_ is applied to the two right FM electrodes, which injects a pure spin current into the metal/Ta_3_*X*_8_/metal layer, the two left FM detector electrodes convert the resulting spin accumulation into a measurable voltage *V*_12_, yielding a nonlocal resistance *R*_12,34_ = *V*_12_/*I*_34_. On the other hand, as spin superconductors, FM Ta_3_*X*_8_ monolayers can realize a spin-current Josephson junction, opening new avenues for developing next-generation spintronic devices. The spin-current Josephson junction is shown in Figure 5B with a spin superconductor/barrier/spin superconductor geometry.

## Resource availability

### Materials availability

The crystal structures of materials are available upon reasonable request.

### Data and code availability

Data are available upon reasonable request.

## Funding and acknowledgments

We thank Prof. Hua Jiang for helpful discussions. This work was supported by the 10.13039/501100012166National Key R&D Program of China (grant no. 2022YFA1403800), the 10.13039/501100001809National Natural Science Foundation of China (grant no. 12188101), and the Center for Materials Genome, China.

## Author contributions

Z.W. conceived and conducted this project. H.S. and J.Y. performed the *GW* + BSE calculations. S.Z. computed the 2D polarizability. H.S., H.W., and Z.W. wrote the paper with input from other authors. The manuscript reflects the contributions of all authors.

## Declaration of interests

The authors declare no competing financial interests.

## Supplemental information

Document S1. Figures S1–S8 and supplemental materials and methods

Document S2. Article plus supplemental information
