# Supplementary material for: Spin-polarized triplet excitonic insulators in Ta3X8 (X = I or Br) monolayers
Source: Innovation (Camb). 2026 Jan 16;7(5):101266. doi: 10.1016/j.xinn.2026.101266 (PMC13147968; doi:10.1016/j.xinn.2026.101266)
Supplement: Document S1. Figures S1–S8 and supplemental materials and methods [file mmc1.pdf]

The Innovation, Volume 7

Supplemental Information

Spin-polarized triplet excitonic insulators in Ta_3X_8 (X = I or Br) monolayers

Haohao Sheng, Jingyu Yao, Sheng Zhang, Quansheng Wu, Zhong Fang, Xi Dai, Hongming Weng, and Zhijun Wang

A. Calculation and Methodology

We carried out first-principles calculations based on density functional theory (DFT) with the projector augmented wave (PAW) method,[1,2] as implemented in the Vienna *ab initio* simulation package (VASP).[3,4] The generalized gradient approximation (GGA) in the form of the Perdew-Burke-Ernzerhof (PBE) functional[5] was employed for the exchange-correlation potential. The kinetic energy cutoff for the plane-wave expansion was set to 330 eV. The Brillouin zone was sampled by the Monkhorst-Pack method in the self-consistent process, with a 6×6×1 **k**-mesh. The thickness of the vacuum layer along c (z) direction was set to $>$ 15 Å. Both lattice parameters and atomic positions were fully relaxed by minimizing the interionic forces below 10^{-3} eV/Å. Spin–orbit coupling (SOC) was included in the electronic structure calculations. The PBE+U method[6–9] and the Heyd–Scuseria–Ernzerhof (HSE06) hybrid functional[10] were employed to check the electronic structure. Based on a previous report for the ferromagnetic (FM) Ta_3I_8 monolayer,[11] we set U at 2 eV. Phonon spectra were obtained using the finite-difference method with a 3×3×1 supercell, as implemented in the Phonopy package.[12] *Ab initio* molecular dynamics (AIMD) simulations were performed using the Nose–Hoover thermostat for 5000 fs with a time step of 1 fs, and the temperature was maintained at 300 K in a 3×3×1 supercell.[13] The generalized momentum matrix element $\boldsymbol{\pi}_{cv}(\boldsymbol{k})$ was calculated using the `vmat=11` function in the VASP2KP package.[14]

In order to consider many-body effects and obtain a more accurate band structure, we carried out the full-frequency single-shot GW calculations (G_0W_0)[15–18] at the PBE level, as implemented in VASP. We also performed partially self-consistent GW calculations ($EVGW_0$) by only iterating G three times to validate the results. In addition, the G_0W_0 calculations at different levels, including PBE+U and HSE06, were performed to further check the results. The GW calculations at the PBE, PBE+U, and HSE06 levels have all demonstrated high reliability in the investigation of excited states.[19–26] Quasi-particle corrections in GW calculations were both **k**-point and band dependent. We employed GW pseudopotentials, which are specifically optimized for accurately treating unoccupied states far above the Fermi level. The same **k**-mesh and a total of 400 bands (305 empty bands) were used. The energy cutoffs for the plane-wave basis set and the response function were 330 eV and 220 eV, respectively, and the soft cutoff for the Coulomb kernel in the response function was 176 eV. The number of frequency grid points was set to 100. After the GW calculations, the band structure was obtained via Wannier interpolation. The maximally localized Wannier functions for the Ta d and I/Br p orbitals were constructed using the Wannier90 package.[27,28]

In order to analyze excitonic properties, we solved the Bethe-Salpeter equation (BSE) under the Tamm-Dancoff approximation using the exact diagonalization algorithm,[29,30] as implemented in VASP. The BSE calculations were performed on top of the GW electronic structure (GW+BSE). Ten valence bands and ten conduction bands were included to build the BSE Hamiltonian. The SOC effect was included in all GW+BSE calculations.

B. Synthesizability and stability

In this section, we carefully consider the synthesizability and stability of Ta_3X_8 (X=I, Br) FM monolayers. First, we perform the convex hull analysis of formation energies for the Ta-I and Ta-Br binary systems. As shown in Figures S1A & D, the energies above the convex hull (E_{hull}) are only 0.01 eV/atom and 0.06 eV/atom for Ta_3I_8 and Ta_3Br_8, respectively. The materials with E_{hull} lower than 0.10 eV/atom are commonly regarded as experimentally accessible candidates.[31,32] Therefore, from this perspective, Ta_3X_8 FM monolayers can be synthesized experimentally. Next, we examine the dynamical and thermal stability via phonon spectra calculations and AIMD simulations. The absence of imaginary frequencies in the phonon dispersion, depicted in Figures S1B & E, confirms that Ta_3I_8 and Ta_3Br_8 FM monolayers are dynamically stable. The AIMD simulations of Ta_3I_8 and Ta_3Br_8 in Figures S1C & F reveal that at 300 K, the free energy fluctuates around a certain value without significant energy decrease over a wide range. Additionally, throughout the 5000 fs simulation, the structure remains intact, indicating thermal stability at room temperature.

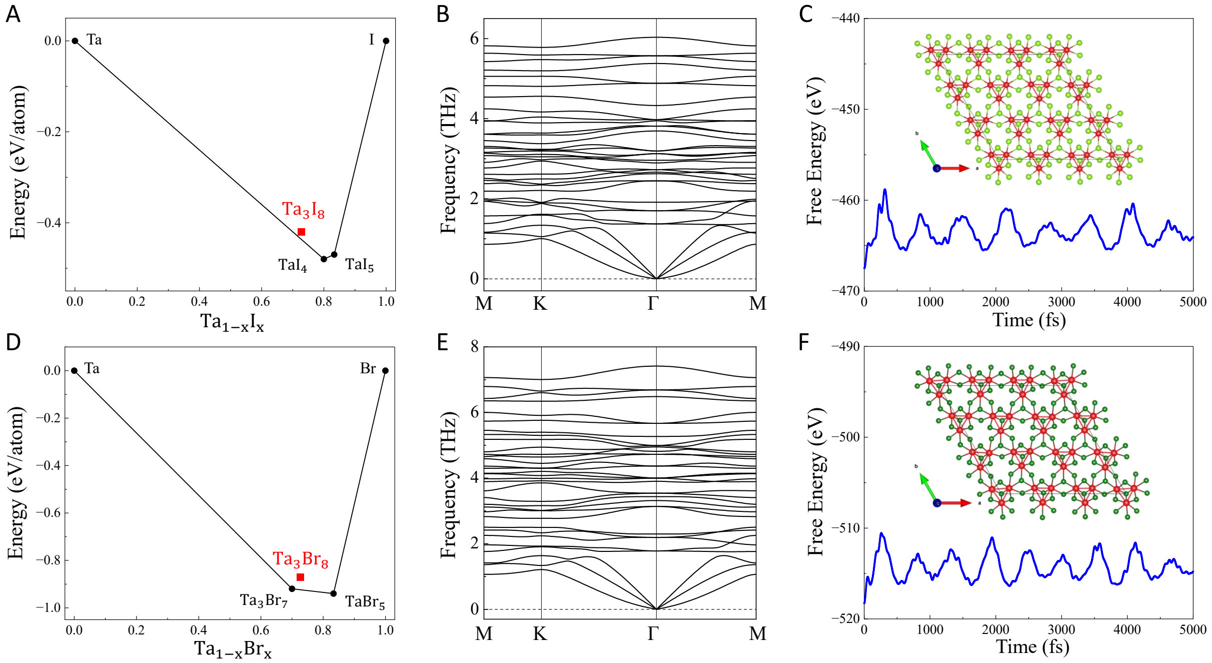

Figure S1. (A, D) Convex hull diagram for the (A) Ta-I and (D) Ta-Br binary systems. The data are sourced from Computational 2D Materials Database.[33,34] (B, E) Phonon spectra of (B) Ta_3I_8 and (E) Ta_3Br_8 FM monolayers. (C, F) AIMD simulations at 300 K of (C) Ta_3I_8 and (F) Ta_3Br_8 FM monolayers. The free energy as a function of simulation time and the structure after the 5000 fs simulation are shown.

C. Convergence tests of GW+BSE calculations at the PBE level

In this section, we thoroughly examine the convergence of GW+BSE calculations at the PBE level. Taking the FM Ta_3I_8 monolayer as an example, we carry out convergence tests of G_0W_0+BSE calculations at the PBE level using a range of different precision parameters, including **k**-mesh, the energy cutoff for the plane-wave basis set (ENCUT), and the total number of bands (NBANDS). When changing ENCUT, the energy cutoff for the response function (ENCUTGW) is set to 2/3 of ENCUT, and the soft cutoff for the Coulomb kernel in the response function (ENCUTGWSOFT) is set to 4/5 of ENCUTGW. The results for the indirect and direct single-particle band gaps (E_g), along with the binding energy (E_b) of the lowest-energy direct exciton, are shown in Figures S2A-C. These results obtained with higher precision parameters show very little variation compared to those presented in the main text, confirming that our G_0W_0+BSE calculations are well converged with respect to these parameters. In addition, we perform partially self-consistent GW calculations ($EVGW_0$) by only iterating G three times. The obtained E_b (1.450 eV) remains larger than E_g (1.393 eV). Therefore, our GW+BSE calculations at the PBE level are highly reliable.

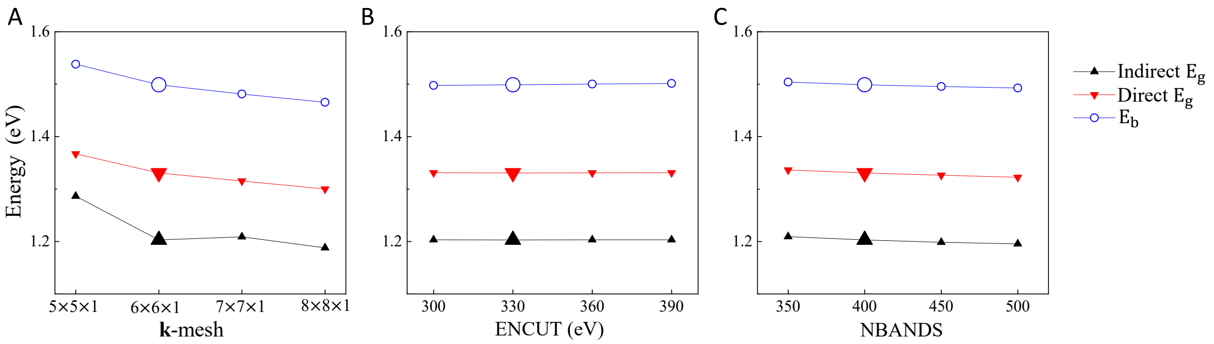

Figure S2. Convergence tests of G_0W_0+BSE calculations at the PBE level for Ta_3I_8 FM monolayer. The results, including the indirect and direct single-particle gaps (E_g) and the exciton binding energy (E_b) of the lowest-energy direct exciton, are shown as functions of (A) **k**-mesh, (B) the energy cutoff for the plane-wave basis set (ENCUT), and (C) the total number of bands (NBANDS). The enlarged data points correspond to the results used in the main text.

D. G_0W_0+BSE calculations at different levels

In this section, we provide a detailed examination of G_0W_0+BSE calculations at different levels, including PBE+U and HSE06. We take the FM Ta_3I_8 monolayer as an example. Figure S3 presents the band structures obtained using the PBE+U and HSE06 methods, as well as the G_0W_0-corrected band structures based on the respective ground states. For each method, the G_0W_0 correction primarily increases E_g without significantly altering the overall band structure. The G_0W_0 band structures at two levels are similar, with the only significant difference being an increase in E_g. Figures S3D & H show the exciton transition energies (E_t) for all direct excitons, obtained from G_0W_0+BSE calculations at the PBE+U and HSE06 levels, respectively. The E_g and the E_b of the lowest-energy exciton from different methods are presented in Figure S4. These results indicate that the E_b consistently exceeds the G_0W_0 E_g for each method. Therefore, the conclusion that the Ta_3I_8 monolayer belongs to a spin-polarized triplet excitonic insulator (EI) state is strongly supported.

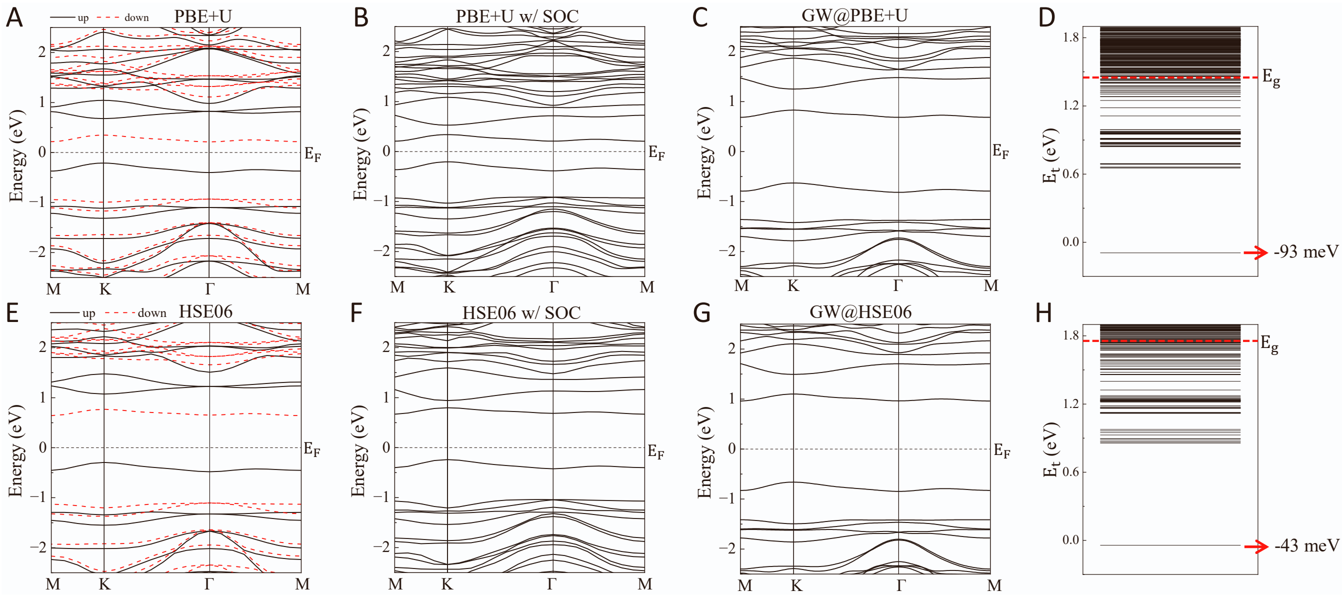

Figure S3. Band structures of Ta_3I_8 FM monolayer calculated using the (A, B) PBE+U and (E, F) HSE06 methods, both without and with SOC. Panels (C) and (G) present the G_0W_0 band structures with SOC at the PBE+U and HSE06 levels, respectively. Exciton transition energy (E_t) spectrum of direct excitons from G_0W_0+BSE calculations at the (D) PBE+U and (H) HSE06 levels. Each horizontal line corresponds to an exciton state. The lowest-energy exciton exhibits a negative E_t.

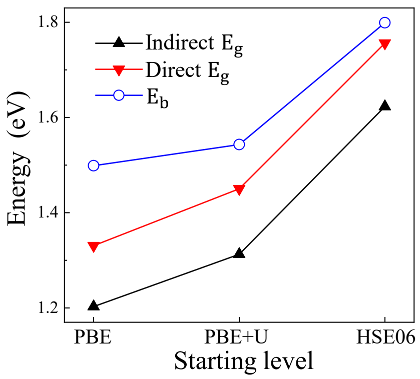

Figure S4. G_0W_0+BSE calculations at the PBE, PBE+U, and HSE06 levels for Ta_3I_8 FM monolayer. The E_g and the E_b of the lowest-energy direct exciton are shown. The E_b consistently exceeds the E_g at each method.

E. External magnetic field, electric field, and strain

In this section, we investigate the influences of external magnetic field, electric field, and strain on the electronic structure for Ta_3I_8 FM monolayer. First, we analyze the influence of an external magnetic field. For this purpose, we calculate the magnetic anisotropy energy. The total energy as a function of magnetization direction is shown in Figure S5. Ta_3I_8 displays out-of-plane (xz-plane) anisotropy, with the energy of the x-directed FM configuration being 0.358 meV per Ta atom lower than that of the z-directed FM configuration. However, the energy differences for the in-plane (xy-plane) directions are less than 0.001 meV. These results indicate that the in-plane FM configuration is the most favorable, and the moment orientation can be effectively tuned by an external magnetic field. We perform G_0W_0+BSE calculations at the PBE level for three FM configurations, where the magnetic moments are aligned parallel to the x, y, and z directions. For these FM configurations, two low-energy flat bands that determine the formation of EI are almost identical, as confirmed by the G_0W_0 band structures in Figure S6. Further BSE calculations show that the E_t values of the lowest-energy direct exciton are -167 meV, -167 meV, and -168 meV, respectively. Therefore, the influence of magnetic direction on the spin-polarized triplet EI state is negligible.

Second, we investigate the effects of electric field and strain on the electronic structure. The spin-polarized band structures under ± 0.5 V/Å vertical electric fields and $\pm 3\%$ biaxial strains are shown in Figures S7B-E. The biaxial strain is defined as $\epsilon = \frac{a-a_0}{a_0} \times 100\%$, where a and a_0 are the lattice constants in the condition of strain and equilibrium. We can observe that, under such electric fields and strains, the two low-energy bands that determine the formation of EI have not undergone any significant changes. Therefore, we conclude that the spin-polarized triplet EI state remains robust under a weak electric field and strain.

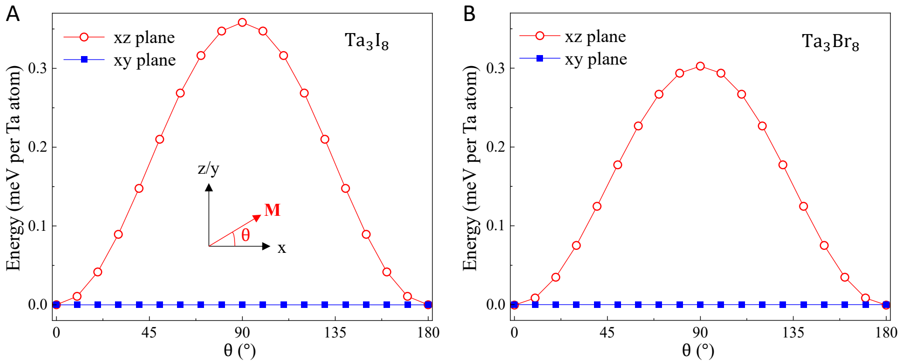

Figure S5. The total energies as a function of magnetization direction in xz and xy planes for (A) Ta_3I_8 and (B) Ta_3Br_8 FM monolayers.

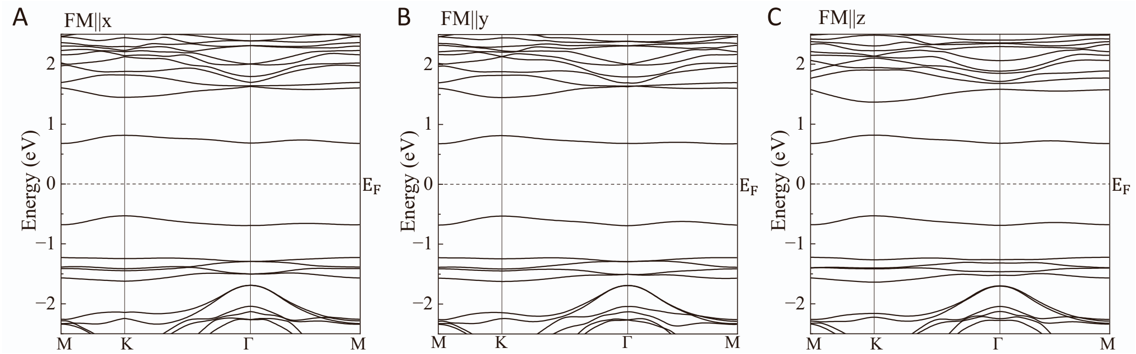

Figure S6. The band structures with SOC from G_0W_0 calculations at the PBE level for Ta_3I_8 FM monolayer, where the magnetic moments are aligned parallel to the (A) x, (B) y, and (C) z directions.

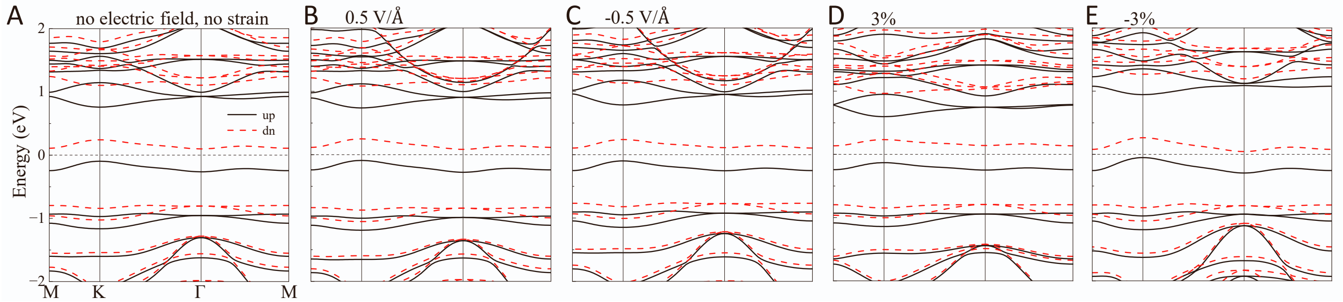

Figure S7. Spin-polarized band structures of Ta_3I_8 FM monolayer, under (A) no vertical electric field and no biaxial strain, (B) 0.5 V/Å vertical electric field, (C) -0.5 V/Å vertical electric field, (D) 3% biaxial strain, and (E) -3% biaxial strain.

F. Electronic structures and low-energy excitons of Ta_3Br_8 monolayer

In this section, we analyze the electronic structures and low-energy excitons of FM Ta_3Br_8 monolayer. Figure S8A presents the spin-polarized band structure and partial densities of states without SOC. The highest valence band (VB) and the lowest conduction band (CB) exhibit opposite spin directions and show minimal energy dispersion. The two low-energy flat bands primarily originate from Ta d_{z^2} orbitals. SOC has little impact on the low-energy flat bands. The same-orbital parity and opposite-spin nature of the band-edge states effectively suppress dielectric screening, promoting the emergence of the EI state.

To analyze low-energy excitons, we perform GW+BSE calculations at the PBE level. As shown in Figure S8B, a more accurate band structure is obtained by many-body single-shot GW calculations (G_0W_0) at the PBE level, where the E_g changes from 0.386 eV (PBE) to 1.722 eV (G_0W_0). Based on the G_0W_0 electronic structure, we solve the BSE using ten VBs and ten CBs. As shown in Figure S8C, the lowest-energy dark direct exciton hosts a negative E_t = -264 meV, indicating that the E_b (1.986 eV) exceeds the E_g. This implies that the Ta_3Br_8 FM monolayer exhibits a many-body EI ground state. We further find that the lowest-energy exciton arises solely from the highest VB and the lowest CB. The spin-flipping in spin-polarized electron-hole transitions can produce spin-polarized triplet excitons with a finite spin moment. Therefore, FM Ta_3Br_8 monolayer is a spin-polarized triplet EI, where the spontaneous BEC of excitons can generate an intriguing spin supercurrent.

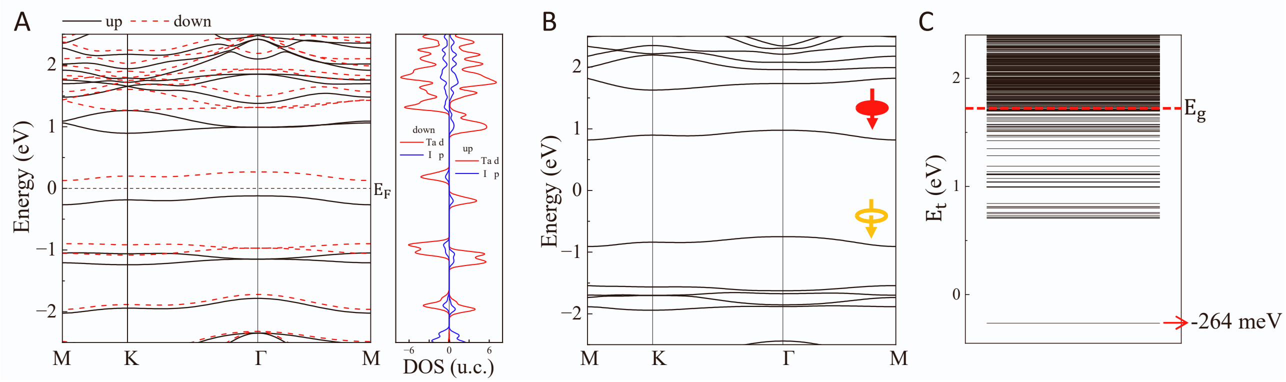

Figure S8. Electronic structure and low-energy excitons of Ta_3Br_8 FM monolayer. (A) The spin-polarized band structure and partial densities of states (DOS) without SOC. (B) The band structure with SOC from G_0W_0 calculations at the PBE level. For the lowest-energy exciton generated by the transition between the two low-energy bands, the orange circled arrow indicates the spin of the hole remaining after excitation, while the red arrow denotes the spin of the excited electron. (C) Exciton transition energy (E_t) spectrum. Each horizontal line corresponds to an exciton state. The lowest-energy exciton exhibits a negative E_t.

REFERENCES

[1] Blöchl P.E. (1994). Projector augmented-wave method. *Phys. Rev. B* **50**:17953. DOI:10.1103/PhysRevB.50.17953
[2] Kresse G. and Joubert D. (1999). From ultrasoft pseudopotentials to the projector augmented-wave method. *Phys. Rev. B* **59**:1758. DOI:10.1103/PhysRevB.59.1758
[3] Kresse G. and Furthmüller J. (1996). Efficiency of ab-initio total energy calculations for metals and semiconductors using a plane-wave basis set. *Comput. Mater. Sci.* **6**:15. DOI:10.1016/0927-0256(96)00008-0
[4] Kresse G. and Furthmüller J. (1996). Efficient iterative schemes for *ab initio* total-energy calculations using a plane-wave basis set. *Phys. Rev. B* **54**:11169. DOI:10.1103/PhysRevB.54.11169
[5] Perdew J.P., Burke K. and Ernzerhof M. (1996). Generalized gradient approximation made simple. *Phys. Rev. Lett.* **77**:3865. DOI:10.1103/PhysRevLett.77.3865
[6] Anisimov V.I., Zaanen J. and Andersen O.K. (1991). Band theory and Mott insulators: Hubbard U instead of Stoner I. *Phys. Rev. B* **44**:943. DOI:10.1103/PhysRevB.44.943
[7] Anisimov V.I., Solovyev I.V., Korotin M.A. *et al.* (1993). Density-functional theory and NiO photoemission spectra. *Phys. Rev. B* **48**:16929. DOI:10.1103/PhysRevB.48.16929
[8] Dudarev S.L., Botton G.A., Savrasov S.Y. *et al.* (1998). Electron-energy-loss spectra and the structural stability of nickel oxide: An LSDA+U study. *Phys. Rev. B* **57**:1505. DOI:10.1103/PhysRevB.57.1505
[9] Anisimov V.I., Aryasetiawan F. and Lichtenstein A.I. (1997). First-principles calculations of the electronic structure and spectra of strongly correlated systems: the LDA+U method. *J. Phys.: Condens. Matter* **9**:767. DOI:10.1088/0953-8984/9/4/002
[10] Krukau A.V., Vydrov O.A., Izmaylov A.F. *et al.* (2006). Influence of the exchange screening parameter on the performance of screened hybrid functionals. *J. Chem. Phys.* **125**:224106. DOI:10.1063/1.2404663
[11] Xing S., Wang B., Zhao T. *et al.* (2024). Independent electrical control of spin and valley degrees in 2D breathing kagome Ta_3I_8 with intrinsic triferroicity. *J. Phys. Chem. Lett.* **15**:6489. DOI:10.1021/acs.jpclett.4c00858
[12] Togo A. and Tanaka I. (2015). First principles phonon calculations in materials science. *Scr. Mater.* **108**:1. DOI:10.1016/j.scriptamat.2015.07.021
[13] Nosé S. (1984). A unified formulation of the constant temperature molecular dynamics methods. *J. Chem. Phys.* **81**:511. DOI:10.1063/1.447334
[14] Zhang S., Sheng H., Song Z. *et al.* (2023). VASP2KP: $k{\cdot}p$ models and Landé g-factors from *ab initio* calculations. *Chin. Phys. Lett.* **40**:127101. DOI:10.1088/0256-307X/40/12/127101
[15] Shishkin M. and Kresse G. (2006). Implementation and performance of the frequency-dependent GW method within the PAW framework. *Phys. Rev. B* **74**:035101. DOI:10.1103/PhysRevB.74.035101
[16] Shishkin M. and Kresse G. (2007). Self-consistent GW calculations for semiconductors and insulators. *Phys. Rev. B* **75**:235102. DOI:10.1103/PhysRevB.75.235102
[17] Fuchs F., Furthmüller J., Bechstedt F. *et al.* (2007). Quasiparticle band structure based on a generalized Kohn-Sham scheme. *Phys. Rev. B* **76**:115109. DOI:10.1103/PhysRevB.76.115109
[18] Shishkin M., Marsman M. and Kresse G. (2007). Accurate quasiparticle spectra from self-consistent GW calculations with vertex corrections. *Phys. Rev. Lett.* **99**:246403. DOI:10.1103/PhysRevLett.99.246403
[19] Jiang Z., Liu Z., Li Y. *et al.* (2017). Scaling universality between band gap and exciton binding energy of two-dimensional semiconductors. *Phys. Rev. Lett.* **118**:266401. DOI:10.1103/PhysRevLett.118.266401
[20] Jiang Z., Li Y., Duan W. *et al.* (2019). Half-excitonic insulator: A single-spin Bose-Einstein condensate. *Phys. Rev. Lett.*

122:236402. DOI:10.1103/PhysRevLett.122.236402
[21] Jiang Z., Lou W., Liu Y. *et al.* (2020). Spin-triplet excitonic insulator: The case of semihydrogenated graphene. *Phys. Rev. Lett.* **124**:166401. DOI:10.1103/PhysRevLett.124.166401
[22] Yao J., Sheng H., Zhang R. *et al.* (2024). Excitonic instability in $Ta_2Pd_3Te_5$ monolayer. *Chin. Phys. Lett.* **41**:097101. DOI:10.1088/0256-307X/41/9/097101
[23] Kharche N., Muckerman J.T. and Hybertsen M.S. (2014). First-principles approach to calculating energy level alignment at aqueous semiconductor interfaces. *Phys. Rev. Lett.* **113**:176802. DOI:10.1103/PhysRevLett.113.176802
[24] Karlický F. and Otyepka M. (2013). Band gaps and optical spectra of chlorographene, fluorographene and graphane from G_0W_0, GW_0 and GW calculations on top of PBE and HSE06 orbitals. *J. Chem. Theory Comput.* **9**:4155. DOI:10.1021/ct400476r
[25] Rodrigues Pela R., Vona C., Lubeck S. *et al.* (2024). Critical assessment of G_0W_0 calculations for 2D materials: the example of monolayer MoS_2. *npj Comput. Mater.* **10**:77. DOI:10.1038/s41524-024-01253-2
[26] Dong S., Chen Y., Qu H. *et al.* (2025). Topological exciton density wave in monolayer WSe_2. *Phys. Rev. Lett.* **134**:066602. DOI:10.1103/PhysRevLett.134.066602
[27] Mostofi A.A., Yates J.R., Pizzi G. *et al.* (2014). An updated version of wannier90: A tool for obtaining maximally-localised Wannier functions. *Comput. Phys. Commun.* **185**:2309. DOI:10.1016/j.cpc.2014.05.003
[28] Marzari N., Mostofi A.A., Yates J.R. *et al.* (2012). Maximally localized Wannier functions: Theory and applications. *Rev. Mod. Phys.* **84**:1419. DOI:10.1103/RevModPhys.84.1419
[29] Albrecht S., Reining L., Del Sole R. *et al.* (1998). *Ab initio* calculation of excitonic effects in the optical spectra of semiconductors. *Phys. Rev. Lett.* **80**:4510. DOI:10.1103/PhysRevLett.80.4510
[30] Rohlfing M. and Louie S.G. (1998). Electron-hole excitations in semiconductors and insulators. *Phys. Rev. Lett.* **81**:2312. DOI:10.1103/PhysRevLett.81.2312
[31] Zeni C., Pinsler R., Zügner D. *et al.* (2025). A generative model for inorganic materials design. *Nature* **639**:624. DOI:10.1038/s41586-025-08628-5
[32] Park H., Onwuli A. and Walsh A. (2025). Exploration of crystal chemical space using text-guided generative artificial intelligence. *Nat. Commun.* **16**:4379. DOI:10.1038/s41467-025-59636-y
[33] Haastrup S., Strange M., Pandey M. *et al.* (2018). The computational 2D materials database: high-throughput modeling and discovery of atomically thin crystals. *2D Mater.* **5**:042002. DOI:10.1088/2053-1583/aacfc1
[34] Gjerding M.N., Taghizadeh A., Rasmussen A. *et al.* (2021). Recent progress of the Computational 2D Materials Database (C2DB). *2D Mater.* **8**:044002. DOI:10.1088/2053-1583/ac1059
