# Supplementary material for: Spin-polarized triplet excitonic insulators in Ta3X8 (X = I or Br) monolayers
Source: Innovation (Camb). 2026 Jan 16;7(5):101266. doi: 10.1016/j.xinn.2026.101266 (PMC13147968; doi:10.1016/j.xinn.2026.101266)
Supplement: Document S2. Article plus supplemental information [file mmc2.pdf]

REPORT Condensed Matter Physics

OPEN ACCESS

Spin-polarized triplet excitonic insulators in Ta_3X_8 (X = I or Br) monolayers

Haohao Sheng,[1,2] Jingyu Yao,[1,2] Sheng Zhang,[1,2] Quansheng Wu,[1,2] Zhong Fang,[1,2] Xi Dai,[3] Hongming Weng,[1,4] and Zhijun Wang[1,4,*]
*Correspondence: wzj@iphy.ac.cn

Received: June 3, 2025; Accepted: January 10, 2026; Published Online: January 16, 2026; https://doi.org/10.1016/j.xinn.2026.101266

© 2026 The Authors. Published by Elsevier Inc. on behalf of Youth Innovation Co., Ltd. This is an open access article under the CC BY license (http://creativecommons.org/licenses/by/4.0/).

www.the-innovation.org

GRAPHICAL ABSTRACT

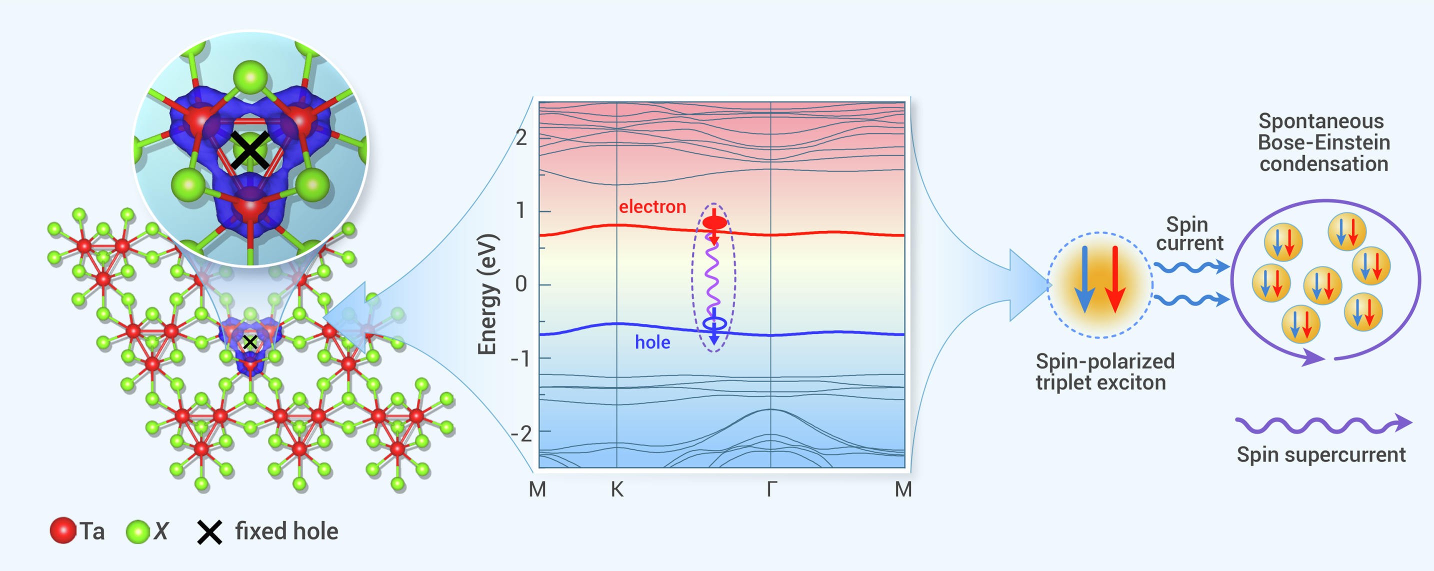

PUBLIC SUMMARY

- Ta_3X_8 (X = I or Br) monolayers exhibit a ferromagnetic insulating ground state.
- Same-orbital parity and opposite-spin nature of band edges yield low two-dimensional polarizability.
- *GW* + BSE calculations reveal a spin-polarized triplet excitonic insulator phase.
- The Bose-Einstein condensation of these excitons can give rise to an intriguing spin supercurrent.

Spin-polarized triplet excitonic insulators in Ta_3X_8 (X = I or Br) monolayers

Haohao Sheng,[1,2] Jingyu Yao,[1,2] Sheng Zhang,[1,2] Quansheng Wu,[1,2] Zhong Fang,[1,2] Xi Dai,[3] Hongming Weng,[1,4] and Zhijun Wang[1,4,*]

[1]Beijing National Laboratory for Condensed Matter Physics, and Institute of Physics, Chinese Academy of Sciences, Beijing 100190, China

[2]University of Chinese Academy of Sciences, Beijing 100049, China

[3]Department of Physics, Hong Kong University of Science and Technology, Clear Water Bay, Hong Kong 999077, China

[4]Condensed Matter Physics Data Center, Chinese Academy of Sciences, Beijing 100190, China

*Correspondence: wzj@iphy.ac.cn

Received: June 3, 2025; Accepted: January 10, 2026; Published Online: January 16, 2026; https://doi.org/10.1016/j.xinn.2026.101266

© 2026 The Authors. Published by Elsevier Inc. on behalf of Youth Innovation Co., Ltd. This is an open access article under the CC BY license (http://creativecommons.org/licenses/by/4.0/).

Citation: Sheng H., Yao J., Zhang S., et al., (2026). Spin-polarized triplet excitonic insulators in Ta_3X_8 (X = I or Br) monolayers. The Innovation **7(5)**, 101266.

Bose-Einstein condensation of spin-polarized triplet excitons can give rise to an intriguing spin supercurrent, providing a direct experimental signature of exciton condensation and offering a non-dissipative channel for information transfer in spintronic devices. In this work, we predict that Ta_3X_8 (X = I or Br) ferromagnetic monolayers are spin-polarized triplet excitonic insulators (EIs), based on the systematic first-principles *GW* calculations coupled with the Bethe-Salpeter equation (*GW* + BSE). The single-particle calculations of spin-polarized band structures reveal that these monolayers are bipolar magnetic semiconductors, where the highest valence band and the lowest conduction band possess opposite spin polarization. The two low-energy bands, primarily originating from Ta d_{z^2} orbitals, are almost flat. The same-orbital parity and opposite-spin nature of the band-edge states effectively suppress dielectric screening, promoting the emergence of the EI state. The *GW* + BSE calculations reveal that the binding energy of the lowest-energy exciton is 1.499 eV for the Ta_3I_8 monolayer and 1.986 eV for the Ta_3Br_8 monolayer, both of which exceed the respective *GW* band gaps, indicating spin-polarized triplet EIs. A wavefunction analysis confirms that the lowest-energy exciton is a tightly bound Frenkel-like state. Our findings establish an ideal material platform for exploring spin-polarized triplet EIs, with promising implications for spintronic applications, such as spin-current Josephson junctions.

INTRODUCTION

Excitons are electron-hole pairs bound by attractive Coulomb interactions. In semiconductors or semimetals, when the exciton binding energy (E_b) exceeds the single-particle band gap (E_g), the spontaneous formation of excitons can cause a renormalization of the single-particle band structure.[1–4] This excitonic instability results in a novel many-body electronic state known as an excitonic insulator (EI). Characterized by the spontaneous Bose-Einstein condensation (BEC) of excitons,[5–7] the time-reversal invariant EI behaves as a perfect insulator for both charge and spin transport. In contrast, a spin-polarized triplet EI is referred to as a spin superconductor.[8,9] Some graphene materials in ferromagnetic (FM) phases have been theoretically predicted to host spin-polarized triplet EIs.[8,10–12] Very recently, the spin-polarized triplet EI state has been experimentally observed in $HfTe_5$ under magnetic fields.[13] However, the intrinsic candidates of the spin-polarized triplet EI remain rare.

To achieve an EI, it is essential to significantly reduce the screening of Coulomb interactions. Dimensionality reduction can weaken electron-hole screening and enhance their binding in two-dimensional (2D) systems.[14,15] Further reduction of screening can be achieved by targeting band-edge states with opposite spin components,[12] the same parity,[16–21] and the same C_{2z} (opposite M_z) symmetry eigenvalues.[22] To date, the experimentally confirmed 2D EIs have been limited to the InAs/GaSb quantum well[23] and the monolayer of 1T'-phase WTe_2.[24,25] On the other hand, the 2D Kagome lattice offers a valuable platform for exploring the interactions between geometry, topology, correlation, multiferroicity, and more.[26–31] In particular, niobium halide clusters are noteworthy due to their breathing Kagome geometry,[32] which supports flat bands.[33–38] This material family features weak interlayer van der Waals interactions, allowing for straightforward thinning down to a 2D limit through mechanical exfoliation.[39,40] Recently, Ta_3X_8 (X = I or Br) monolayers have been predicted to be stable 2D intrinsic multiferroic semiconductors with the coexistence of FM, ferroelectric, and ferrovalley orders.[41–43] However, their exciton properties are still unrevealed.

In this work, we predict that Ta_3X_8 monolayers are spin-polarized triplet EIs, as demonstrated by systematic first-principles *GW* calculations coupled with the Bethe-Salpeter equation (*GW* + BSE). The single-particle calculations reveal that these monolayers are bipolar magnetic semiconductors (BMSs), where the highest valence band (VB) and the lowest conduction band (CB) are fully spin-polarized in opposite spin directions. Moreover, the two low-energy bands from the Ta d_{z^2} orbitals exhibit minimal energy dispersion, forming flat bands. The characteristics of same-orbital parity and opposite-spin band-edge states effectively suppress band-edge transitions and dielectric screening, facilitating the realization of the EI state. The *GW* + BSE calculations show that the *GW* band gap is 1.331 (1.722) eV, while the E_b of the lowest-energy exciton reaches 1.499 (1.986) eV for the Ta_3I_8 (Ta_3Br_8) monolayer, exceeding the corresponding *GW* band gap. A wavefunction analysis confirms that the lowest-energy exciton is a spin-polarized triplet state. The results indicate that FM Ta_3X_8 monolayers are spin-polarized triplet EIs, where spontaneous exciton BEC can generate an intriguing spin supercurrent.

MATERIALS AND METHODS

We carried out first-principles calculations based on density functional theory with the projector augmented wave method,[44,45] as implemented in the Vienna *Ab initio* Simulation Package.[46,47] The generalized gradient approximation in the form of the Perdew-Burke-Ernzerhof (PBE) functional[48] was used for the exchange-correlation potential. Phonon spectra were obtained using the finite-difference method, as implemented in the Phonopy package.[49] The full-frequency single-shot *GW* calculations (G_0W_0)[50–53] at the PBE level were performed to obtain a more accurate band structure. In order to analyze excitonic properties, we solved the BSE[54,55] on top of the *GW* electronic structure (*GW* + BSE). More details of the calculation methods are provided in section A of the supplemental materials and methods.

RESULTS AND DISCUSSION

Crystal and electronic structures

The Ta_3X_8 monolayers form a breathing Kagome lattice with space group $P3m1$, as illustrated in Figure 1A. The structure is derived by removing a Ta atom from the 2 × 2 supercell of the 1T-phase TaX_2 and introducing a breathing distortion, resulting in Ta-trimer clusters in the Kagome lattice. First-principles single-particle calculations show that they exhibit an FM ground state with a total magnetic moment of 1 μ_B per unit cell. Since Ta_3X_8 monolayers have the same properties, we take the Ta_3I_8 monolayer as an example in the main text (see the results for the Ta_3Br_8 monolayer in section F of the supplemental materials and methods). The phonon dispersion of the FM state of the Ta_3I_8 monolayer is presented in Figure 1B, indicating that it is dynamically stable. Detailed analysis of structural stability can be found in section B of the supplemental materials and methods. In the spin-polarized band structure shown in Figure 1C, the highest VB and the lowest CB exhibit opposite spin directions, a hallmark feature of BMSs.[56–58] Moreover, the two low-energy bands are almost flat (with ~0.1 eV bandwidths), suggesting strong interelectronic correlations within the system. Considering spin-orbit coupling (SOC), the band structure is shown in Figure 2A, demonstrating that it has little impact on the two flat bands. From the partial densities of states in Figure 1D and the orbital-resolved band structure in Figure 2A, we find that the low-energy flat bands are mainly from Ta d_{z^2} orbitals.

www.the-innovation.org

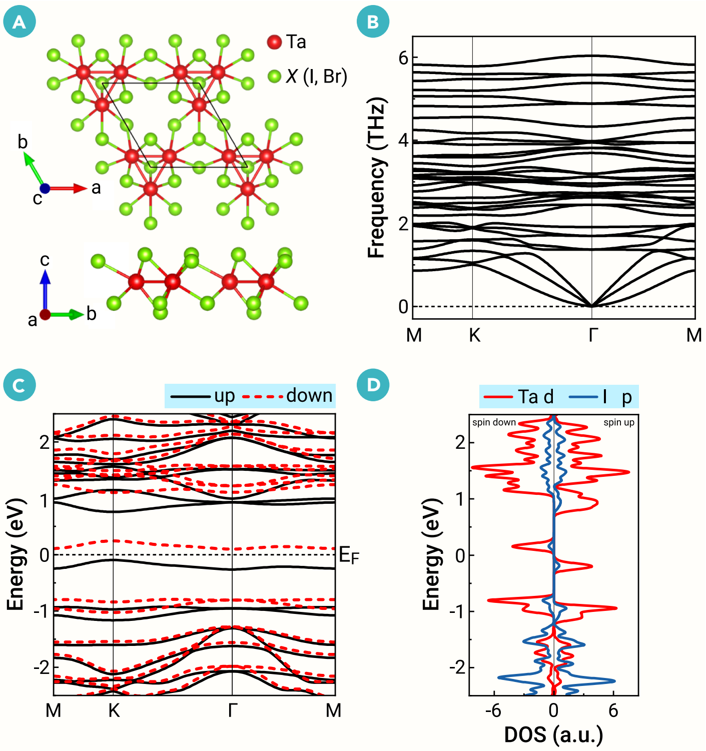

Figure 1. Crystal and electronic structures The (A) crystal structure, (B) phonon spectra, and (C and D) electronic band structures of Ta_3I_8 monolayer. The unit cell is outlined in black in (A). (C) The spin-polarized band structure and (D) partial densities of states (DOSs) without spin-orbit coupling (SOC). Black and red dotted lines represent the spin-up and spin-down bands, respectively.

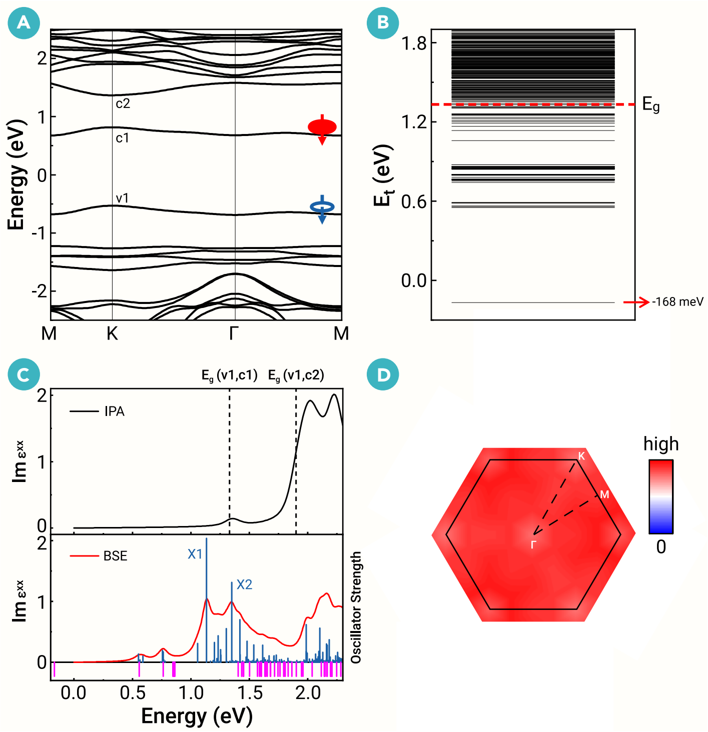

Figure 3. Low-energy direct excitons of Ta_3I_8 monolayer (A) The G_0W_0 band structure with SOC. The lowest-energy exciton generated by the transition between the v1 and c1 bands is shown. The blue arrow with a circle denotes the spin of the hole left behind after excitation, and the red arrow denotes the spin of the electron. (B) Exciton transition energy (E_t) spectrum. Each horizontal line corresponds to an exciton state. The lowest-energy exciton exhibits a negative E_t. (C) (Top) Imaginary part of the dielectric function under the independent-particle approximation (IPA), i.e., ignoring electron-hole interactions. (Bottom) Imaginary part of the dielectric function (left axis) and exciton oscillator strength (right axis) from the BSE calculation. The bright excitons are depicted by blue vertical lines, with the height indicating the oscillator strength, while the dark excitons are shown by the pink vertical lines under the x axis, whose strengths are less than 5×10^{-6} that of the brightest exciton. (D) Exciton wavefunction in reciprocal space for the lowest-energy exciton. Its substantial delocalization in reciprocal space corresponds to a high localization in real space.

Forbidden band-edge transition

In 2D materials, a previous study has shown that E_b often scales with E_g, typically as $E_b \approx E_g/4$.[59] Therefore, to achieve a 2D EI, one has to break this relationship in the materials by reducing the dielectric constant (screening of Coulomb interactions). For this purpose, we directly compute the 2D polarizability α_{2D} (2D static dielectric constant)[59,60] in the random phase approximation approach and in the absence of the local field effect,[61]

$$\alpha_{2D}^{\beta\beta} = L\frac{\varepsilon^{\beta\beta}-1}{4\pi} = \frac{1}{N_{\boldsymbol{k}}}\sum_{c,v,\boldsymbol{k}}\tilde{\alpha}_{2D;cv}^{\beta\beta}(\boldsymbol{k}),$$

$$\tilde{\alpha}_{2D;cv}^{\beta\beta}(\boldsymbol{k}) = \frac{1}{4\pi S}\frac{8\pi e^2\hbar^2}{m_e^2}\frac{\pi_{cv}^{\beta}(\boldsymbol{k})\pi_{vc}^{\beta}(\boldsymbol{k})}{\left(E_{c,\boldsymbol{k}} - E_{v,\boldsymbol{k}}\right)^3}, \qquad \text{(Equation 1)}$$

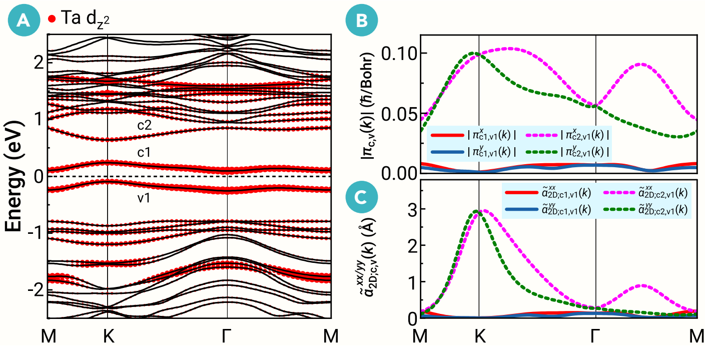

Figure 2. Fatband structure and 2D polarizability (A) The orbital-resolved band structure with SOC of Ta_3I_8 monolayer. The size of the red dots represents the weight of the Ta d_{z^2} orbitals. (B) The modulus of the generalized momentum matrix elements $\boldsymbol{\pi}_{c1(c2),v1}(\boldsymbol{k})$ along the high-symmetry paths. (C) 2D polarizability $\tilde{\alpha}_{2D;c1(c2),v1}^{xx/yy}(\boldsymbol{k})$ along the high-symmetry paths.

where L denotes the vacuum thickness in the z direction, S is the area of the unit cell in the xy plane, $\varepsilon^{\beta\beta}$ denotes the static dielectric constant of the 3D system, $N_{\boldsymbol{k}}$ is the number of $\boldsymbol{k}$ points, and $\boldsymbol{\pi}_{cv}(\boldsymbol{k})$ represents the generalized momentum matrix including SOC, given by $\boldsymbol{\pi}_{cv}(\boldsymbol{k}) \equiv \langle u_{c,\boldsymbol{k}} \left| \hat{\boldsymbol{p}} + \frac{1}{2mc^2}(\hat{\boldsymbol{s}} \times \nabla V(\boldsymbol{r})) \right| u_{v,\boldsymbol{k}} \rangle$. Here, $\hat{\boldsymbol{p}}$ is the momentum operator, $V(\boldsymbol{r})$ is the potential in the crystal, $\hat{\boldsymbol{s}}$ is the spin momentum operator, and $u_{c,\boldsymbol{k}}$ and $u_{v,\boldsymbol{k}}$ refer to the periodic parts of CB and VB Bloch states, respectively. Calculated by the VASP2KP,[62] the obtained $\boldsymbol{\pi}_{c1(c2),v1}(\boldsymbol{k})$ and $\tilde{\alpha}_{2D;c1(c2),v1}^{xx/yy}(\boldsymbol{k})$ along the high-symmetry paths are presented in Figures 2B and 2C, where v1 and c1(c2) are the highest VB and the lowest (second-lowest) CB, respectively. One can see that $\boldsymbol{\pi}_{c1,v1}(\boldsymbol{k})$ is extremely low, less than 0.01 $\hbar$/Bohr. This result implies that optical transitions between the v1 and c1 bands are essentially forbidden; therefore, these bands make a negligible contribution to dielectric screening, with $\tilde{\alpha}_{2D;c1,v1}$ only 0.07 Å. As a result, the total 2D polarizability (α_{2D} = 5.20 Å) is very low, much smaller than that of conventional 2D materials with similar band gaps ($\alpha_{2D} \sim 30$ Å).[59] These results clearly indicate a significant suppression of dielectric screening due to the low-energy v1 and c1 bands, which can decouple E_b from E_g and significantly enhance E_b, enabling the realization of the EI state in the monolayer. We attribute the minor value of $\tilde{\alpha}_{2D;c1,v1}$ to the following reasons. First, the low-energy flat Ta d orbital bands suggest that Ta atoms maintain good localized atomic characteristics. According to the selection rules for atomic orbital transitions, the low-energy d-d transitions are parity forbidden. Second, in systems with weak SOC, electric-dipole transitions adhere to the spin selection rule.

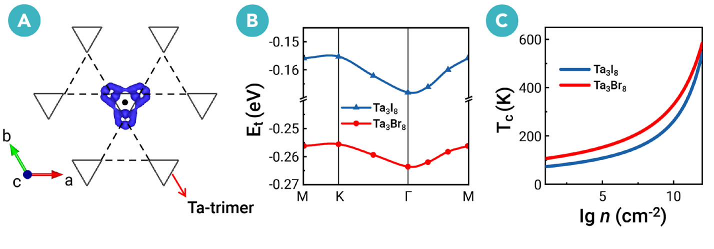

Figure 4. Exciton real-space wave function, dispersion, and critical temperature (A) Exciton wavefunction in real space of the lowest-energy exciton for Ta_3I_8 monolayer. Only the Ta-breathing Kagome lattice and a portion of the $6 \times 6 \times 1$ supercell are shown. The contour plot (blue) is the probability density of locating the bound electron once the hole position is fixed (black dot). (B) Exciton dispersion of the lowest-energy exciton along the high-symmetry paths for Ta_3X_8 monolayers. (C) The critical temperature (T_c) of exciton condensation as a function of the exciton density (lg$n$) for Ta_3X_8 monolayers.

Excitons with direct transition

To analyze low-energy excitons, we perform *GW* + BSE calculations at the PBE level (section C of the supplemental materials and methods). As shown in Figure 3A, a more accurate band structure is obtained by many-body G_0W_0 calculations, where the E_g changes from 0.334 (PBE) to 1.331 (G_0W_0) eV. Based on the G_0W_0 electronic structure, we solve the BSE using ten VBs and ten CBs. Figure 3B shows the exciton transition energies (E_t) for all direct excitons. The lowest-energy exciton exhibits a negative E_t = −168 meV, indicating that the E_b exceeds the E_g. This implies that the Ta_3I_8 monolayer exhibits a many-body EI ground state. This result is also checked by the *GW* + BSE calculations at the PBE+U and HSE06 levels (section D of the supplemental materials and methods). The lowest-energy exciton is identified as a dark state due to its extremely low oscillator strength. The imaginary part of the frequency-dependent dielectric function under the independent-particle approximation (IPA) is shown in the top image of Figure 3C. We can see very weak optical absorption at the band edge (E_g(v1,c1)) because the band-edge transition is effectively suppressed, which is consistent with the low $\tilde{\alpha}_{2D;c1,v1}$. The imaginary part of the frequency-dependent dielectric function from the BSE calculations is presented in the bottom image of Figure 3C. Compared to the IPA, two prominent optical absorption peaks emerge, originating from two bright excitons (X1 and X2). These exciton wavefunctions show that X1 and X2 excitons arise from optically allowed transitions between the v1 and c2 bands.

We primarily focus on the lowest-energy exciton. Figure 3D illustrates its reciprocal-space wavefunction, which extends across the entire Brillouin zone (BZ). Its substantial delocalization in reciprocal space corresponds to a high localization in real space, resembling a tightly bound Frenkel-like exciton. As shown in Figure 4A, when a hole is introduced at the center of the Ta trimer, the bound electron becomes localized exclusively on the nearest Ta atoms. We further find that the lowest-energy exciton arises solely from the v1 and c1 bands. The spin-flipping in spin-polarized electron-hole transitions can produce spin-polarized triplet excitons with a finite spin moment ($s_z = \hbar$). This spin-polarized triplet EI state remains robust under weak external fields (section E of the supplemental materials and methods).

Exciton dispersion

The **q**-dependent lowest E_t of Ta_3X_8 monolayers along the high-symmetry paths is shown in Figure 4B. The E_t remains negative across all **q** values, which indicates that the EI state is accessible for all momenta in the full BZ. Although Ta_3X_8 monolayers host an indirect E_g, the direct exciton with **q** = 0 is energetically more stable than those with **q** ≠ 0. This indirect-to-direct transition crossover between the single-particle band dispersion and the exciton dispersion is related to nonlocal dielectric screening.[12] In addition, Ta_3X_8 monolayers exhibit small exciton dispersion with bandwidths narrower than 13 meV, a direct consequence of their electronic flat bands. The momentum-dependent dispersion of excitons may be experimentally probed using momentum-resolved electron energy loss spectroscopy or resonant inelastic X-ray spectroscopy.[63–67]

Critical temperature

Unlike parabolic bands, the low-energy flat bands can make excitons condense into an ideal form, such as one-body bosons.[68] For spontaneously formed excitons treated as an ideal Bose gas,[18,69–71] the condensation critical temperature (T_c) as a function of the exciton density (n) is given by

$$n = -\frac{mk_BT_c}{2\pi\hbar^2}\ln\left(1 - e^{-|E_t|/(k_BT_c)}\right), \qquad \text{(Equation 2)}$$

where exciton mass $m = m_e + m_h$ is obtained by fitting the band dispersion. Experimentally, spontaneous exciton condensation has been realized at a density of up to 10^{11}–10^{12} cm $^{-2}$ in the 1*T′*-WTe_2 monolayer.[24,25] As presented in Figure 4C, with a density $n = 10^{11}$ cm^{-2}, the T_c values of Ta_3I_8 and Ta_3Br_8 EIs are 355 and 420 K, respectively. These results imply that the EI state may remain stable at room temperature. This contrasts sharply with typical Bardeen-Cooper-Schrieffer (BCS) superconductors, whose transition temperatures are usually only 1–10 K. The reason is that strong electron-hole pairing in EI is driven by direct Coulomb attraction, while weak electron pairing in BCS superconductors is mediated by phonons.

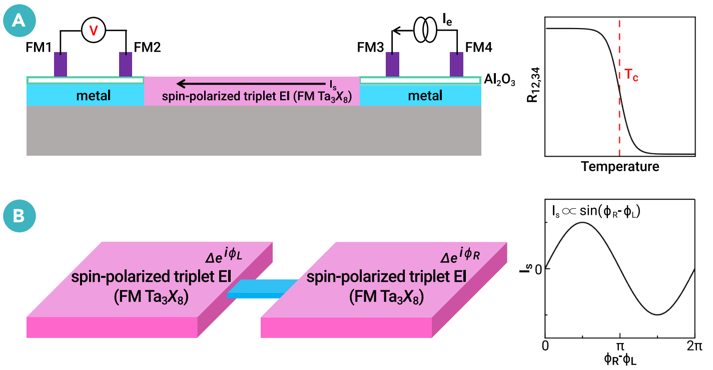

Figure 5. Four-terminal device and spin-current Josephson junction (A) Schematic diagram of the four-terminal device to detect spin supercurrent as a direct experimental signature of spin-polarized triplet exciton condensation. The measurable nonlocal resistance $R_{12,34}$ as a function of temperature is shown. (B) Schematic diagram of the spin-current Josephson junction with a spin-superconductor/barrier/spin-superconductor geometry. At equilibrium, the spin supercurrent I_s as a function of the phase difference between the right and left spin superconductors ($\phi_R - \phi_L$) is shown.

CONCLUSION

In summary, we predict that Ta_3X_8 FM monolayers are spin-polarized triplet EIs, based on systematic first-principles *GW* + BSE calculations. Single-particle calculations reveal that these monolayers are intrinsic BMSs. The calculated E_b of the lowest-energy exciton exceeds the E_g, indicating that Ta_3X_8 monolayers belong to a spin-polarized triplet EI ground state. Analysis of the exciton wavefunction further reveals that the lowest-energy exciton is a tightly bound Frenkel-like state. The estimated T_c of exciton condensation could reach room temperature at a proper exciton density. This study offers an ideal material platform for developing next-generation spintronic devices with spin-manipulation capabilities.

Unlike the time-reversal invariant EIs,[23–25] which are perfect insulators for both charge and spin transport, the spin-polarized triplet EIs in FM Ta_3X_8 monolayers have a finite spin moment, giving rise to an intriguing spin supercurrent. On the one hand, we suggest a four-terminal device[8] in Figure 5A to detect the spin supercurrent as a direct experimental signature of this exciton condensation. After a current I_{34} is applied to the two right FM electrodes, which injects a pure spin current into the metal/Ta_3X_8/metal layer, the two left FM detector electrodes convert the resulting spin accumulation into a measurable voltage V_{12}, yielding a nonlocal resistance $R_{12,34} = V_{12}/I_{34}$. On the other hand, as spin superconductors, FM Ta_3X_8 monolayers can realize a spin-current Josephson junction, opening new avenues for developing next-generation spintronic devices. The spin-current Josephson junction is shown in Figure 5B with a spin superconductor/barrier/spin superconductor geometry.

RESOURCE AVAILABILITY

Materials availability

The crystal structures of materials are available upon reasonable request.

www.the-innovation.org

Data and code availability

Data are available upon reasonable request.

FUNDING AND ACKNOWLEDGMENTS

We thank Prof. Hua Jiang for helpful discussions. This work was supported by the National Key R&D Program of China (grant no. 2022YFA1403800), the National Natural Science Foundation of China (grant no. 12188101), and the Center for Materials Genome, China.

AUTHOR CONTRIBUTIONS

Z.W. conceived and conducted this project. H.S. and J.Y. performed the *GW* + BSE calculations. S.Z. computed the 2D polarizability. H.S., H.W., and Z.W. wrote the paper with input from other authors. The manuscript reflects the contributions of all authors.

DECLARATION OF INTERESTS

The authors declare no competing financial interests.

SUPPLEMENTAL INFORMATION

It can be found online at https://doi.org/10.1016/j.xinn.2026.101266.

REFERENCES

1. Jérome, D., Rice, T.M. and Kohn, W. (1967). Excitonic insulator. *Phys. Rev.* **158**:462–475. DOI:10.1103/PhysRev.158.462
2. Kohn, W. (1967). Excitonic phases. *Phys. Rev. Lett.* **19**:439–442. DOI:10.1103/PhysRevLett.19.439
3. Halperin, B.I. and Rice, T.M. (1968). Possible anomalies at a semimetal-semiconductor transistion. *Rev. Mod. Phys.* **40**:755–766. DOI:10.1103/RevModPhys.40.755
4. Mott, N.F. (1961). The transition to the metallic state. *The Philosophical Magazine: A Journal of Theoretical Experimental and Applied Physics* **6**:287–309. DOI:10.1080/14786436108243318
5. Kohn, W. and Sherrington, D. (1970). Two kinds of bosons and bose condensates. *Rev. Mod. Phys.* **42**:1–11. DOI:10.1103/RevModPhys.42.1
6. Eisenstein, J.P. and Macdonald, A.H. (2004). Bose–Einstein condensation of excitons in bilayer electron systems. *Nature* **432**:691–694. DOI:10.1038/nature03081
7. Kogar, A., Rak, M.S., Vig, S. et al. (2017). Signatures of exciton condensation in a transition metal dichalcogenide. *Science* **358**:1314–1317. DOI:10.1126/science.aam6432
8. Sun, Q.f., Jiang, Z.t., Yu, Y. et al. (2011). Spin superconductor in ferromagnetic graphene. *Phys. Rev. B* **84**:214501. DOI:10.1103/PhysRevB.84.214501
9. Bao, Z.q., Xie, X.C. and Sun, Q.f. (2013). Ginzburg–landau-type theory of spin superconductivity. *Nat. Commun.* **4**:2951. DOI:10.1038/ncomms3951
10. Liu, H., Jiang, H., Xie, X.C. et al. (2012). Spontaneous spin-triplet exciton condensation in ABC-stacked trilayer graphene. *Phys. Rev. B* **86**:085441. DOI:10.1103/PhysRevB.86.085441
11. Li, S., Ren, Y.H., Li, A.L. et al. (2024). Spontaneous spin superconductor state in ABCA-stacked tetralayer graphene. *Phys. Rev. B* **110**:174512. DOI:10.1103/PhysRevB.110.174512
12. Jiang, Z., Lou, W., Liu, Y. et al. (2020). Spin-triplet excitonic insulator: The case of semihydrogenated graphene. *Phys. Rev. Lett.* **124**:166401. DOI:10.1103/PhysRevLett.124.166401
13. Liu, J., Subramanyan, V., Welser, R. et al. (2025). Possible spin-triplet excitonic insulator in the ultra-quantum limit of $HfTe_5$. *Phys. Rev. Lett.* **135**:046601. DOI:10.1103/bj2n-4k2w
14. Wang, G., Chernikov, A., Glazov, M.M. et al. (2018). Colloquium: Excitons in atomically thin transition metal dichalcogenides. *Rev. Mod. Phys.* **90**:021001. DOI:10.1103/RevModPhys.90.021001
15. Chernikov, A., Berkelbach, T.C., Hill, H.M. et al. (2014). Exciton binding energy and nonhydrogenic Rydberg series in monolayer WS_2. *Phys. Rev. Lett.* **113**:076802. DOI:10.1103/PhysRevLett.113.076802
16. Jiang, Z., Li, Y., Zhang, S. et al. (2018). Realizing an intrinsic excitonic insulator by decoupling exciton binding energy from the minimum band gap. *Phys. Rev. B* **98**:081408(R). DOI:10.1103/PhysRevB.98.081408
17. Dong, S. and Li, Y. (2021). Excitonic instability and electronic properties of AlSb in the two-dimensional limit. *Phys. Rev. B* **104**:085133. DOI:10.1103/PhysRevB.104.085133
18. Dong, S. and Li, Y. (2023). Robust high-temperature topological excitonic insulator of transition-metal carbide MXenes. *Phys. Rev. B* **107**:235147. DOI:10.1103/PhysRevB.107.235147
19. Yang, H., Zeng, J., Shao, Y. et al. (2024). Spin-triplet topological excitonic insulators in two-dimensional materials. *Phys. Rev. B* **109**:075167. DOI:10.1103/PhysRevB.109.075167
20. Jiang, Z., Li, Y., Duan, W. et al. (2019). Half-excitonic insulator: A single-spin Bose-Einstein condensate. *Phys. Rev. Lett.* **122**:236402. DOI:10.1103/PhysRevLett.122.236402
21. Dong, S., Chen, Y., Qu, H. et al. (2025). Topological exciton density wave in monolayer WSe_2. *Phys. Rev. Lett.* **134**:066602. DOI:10.1103/PhysRevLett.134.066602
22. Yao, J., Sheng, H., Zhang, R. et al. (2024). Excitonic instability in $Ta_2Pd_3Te_5$ monolayer. *Chin. Phys. Lett.* **41**:097101. DOI:10.1088/0256-307X/41/9/097101
23. Du, L., Li, X., Lou, W. et al. (2017). Evidence for a topological excitonic insulator in InAs/GaSb bilayers. *Nat. Commun.* **8**:1971. DOI:10.1038/s41467-017-01988-1
24. Jia, Y., Wang, P., Chiu, C.L. et al. (2022). Evidence for a monolayer excitonic insulator. *Nat. Phys.* **18**:87–93. DOI:10.1038/s41567-021-01422-w
25. Sun, B., Zhao, W., Palomaki, T. et al. (2022). Evidence for equilibrium exciton condensation in monolayer WTe_2. *Nat. Phys.* **18**:94–99. DOI:10.1038/s41567-021-01427-5
26. Ghimire, N.J. and Mazin, I.I. (2020). Topology and correlations on the kagome lattice. *Nat. Mater.* **19**:137–138. DOI:10.1038/s41563-019-0589-8
27. Teng, X., Chen, L., Ye, F. et al. (2022). Discovery of charge density wave in a kagome lattice antiferromagnet. *Nature* **609**:490–495. DOI:10.1038/s41586-022-05034-z
28. Yin, J.X., Lian, B. and Hasan, M.Z. (2022). Topological kagome magnets and superconductors. *Nature* **612**:647–657. DOI:10.1038/s41586-022-05516-0
29. Deng, J., Zhang, R., Xie, Y. et al. (2023). Two elementary band representation model, fermi surface nesting, and surface topological superconductivity in AV_3Sb_5 (A = K, Rb, Cs). *Phys. Rev. B* **108**:115123. DOI:10.1103/PhysRevB.108.115123
30. Xie, Y., Ji, K., He, J. et al. (2025). Manipulation of topology by electric field in breathing kagome lattice. *Phys. Rev. Lett.* **135**:056701. DOI:10.1103/29ht-pwyt
31. Li, C., Xie, Y., Li, Y. et al. (2025). Generation and annihilation of local magnetic moments by an electric field. *Nano Lett.* **25**:12762–12768. DOI:10.1021/acs.nanolett.5c03407
32. Magonov, S.N., Zoennchen, P., Rotter, H. et al. (1993). Scanning tunneling and atomic force microscopy study of layered transition metal halides Nb_3X_8 (X = Cl, Br, I). *J. Am. Chem. Soc.* **115**:2495–2503. DOI:10.1021/ja00059a053
33. Regmi, S., Fernando, T., Zhao, Y. et al. (2022). Spectroscopic evidence of flat bands in breathing kagome semiconductor Nb_3I_8. *Commun. Mater.* **3**:100. DOI:10.1038/s43246-022-00318-3
34. Regmi, S., Sakhya, A.P., Fernando, T. et al. (2023). Observation of flat and weakly dispersing bands in the van der Waals semiconductor Nb_3Br_8 with breathing kagome lattice. *Phys. Rev. B* **108**:L121404. DOI:10.1103/PhysRevB.108.L121404
35. Sun, Z., Zhou, H., Wang, C. et al. (2022). Observation of topological flat bands in the kagome semiconductor Nb_3Cl_8. *Nano Lett.* **22**:4596–4602. DOI:10.1021/acs.nanolett.2c00778
36. Liu, H., Meng, S. and Liu, F. (2021). Screening two-dimensional materials with topological flat bands. *Phys. Rev. Mater.* **5**:084203. DOI:10.1103/PhysRevMaterials.5.084203
37. Gao, S., Zhang, S., Wang, C. et al. (2023). Discovery of a single-band Mott insulator in a van der Waals flat-band compound. *Phys. Rev. X* **13**:041049. DOI:10.1103/PhysRevX.13.041049
38. Zhang, Y., Gu, Y., Weng, H. et al. (2023). Mottness in two-dimensional van der Waals Nb_3X_8 monolayers (X=Cl, Br, and I). *Phys. Rev. B* **107**:035126. DOI:10.1103/PhysRevB.107.035126
39. Oh, S., Choi, K.H., Chae, S. et al. (2020). Large-area synthesis of van der Waals two-dimensional material Nb_3I_8 and its infrared detection applications. *J. Alloys Compd.* **831**:154877. DOI:10.1016/j.jallcom.2020.154877
40. Kim, B.J., Jeong, B.J., Oh, S. et al. (2019). Structural and electrical properties of Nb_3I_8 layered crystal. *Physica Rapid Research Ltrs.* **13**:1800448. DOI:10.1002/pssr.201800448
41. Deng, J., Pan, J., Zhang, Y. et al. (2022). Screening and design of bipolar magnetic-semiconducting monolayers and heterostructures. *ACS Appl. Electron. Mater.* **4**:3232–3239. DOI:10.1021/acsaelm.2c00464
42. Xing, S., Wang, B., Zhao, T. et al. (2024). Independent electrical control of spin and valley degrees in 2D breathing kagome Ta_3I_8 with intrinsic triferroicity. *J. Phys. Chem. Lett.* **15**:6489–6495. DOI:10.1021/acs.jpclett.4c00858
43. Lu, J., Chen, H., Zhao, X. et al. (2024). Chiral breathing-valley locking in two-dimensional kagome lattice Ta_3I_8. *Appl. Phys. Lett.* **124**:072101. DOI:10.1063/5.0193656
44. Blöchl, P.E. (1994). Projector augmented-wave method. *Phys. Rev. B* **50**:17953–17979. DOI:10.1103/PhysRevB.50.17953
45. Kresse, G. and Joubert, D. (1999). From ultrasoft pseudopotentials to the projector augmented-wave method. *Phys. Rev. B* **59**:1758–1775. DOI:10.1103/PhysRevB.59.1758
46. Kresse, G. and Furthmüller, J. (1996). Efficiency of ab-initio total energy calculations for metals and semiconductors using a plane-wave basis set. *Comput. Mater. Sci.* **6**:15–50. DOI:10.1016/0927-0256(96)00008-0
47. Kresse, G. and Furthmüller, J. (1996). Efficient iterative schemes for ab initio total-energy calculations using a plane-wave basis set. *Phys. Rev. B* **54**:11169–11186. DOI:10.1103/PhysRevB.54.11169
48. Perdew, J.P., Burke, K. and Ernzerhof, M. (1996). Generalized gradient approximation made simple. *Phys. Rev. Lett.* **77**:3865–3868. DOI:10.1103/PhysRevLett.77.3865
49. Togo, A. and Tanaka, I. (2015). First principles phonon calculations in materials science. *Scr. Mater.* **108**:1–5. DOI:10.1016/j.scriptamat.2015.07.021
50. Shishkin, M. and Kresse, G. (2006). Implementation and performance of the frequency-dependent *GW* method within the PAW framework. *Phys. Rev. B* **74**:035101. DOI:10.1103/PhysRevB.74.035101
51. Shishkin, M. and Kresse, G. (2007). Self-consistent *GW* calculations for semiconductors and insulators. *Phys. Rev. B* **75**:235102. DOI:10.1103/PhysRevB.75.235102
52. Fuchs, F., Furthmüller, J., Bechstedt, F. et al. (2007). Quasiparticle band structure based on a generalized Kohn-Sham scheme. *Phys. Rev. B* **76**:115109. DOI:10.1103/PhysRevB.76.115109
53. Shishkin, M., Marsman, M. and Kresse, G. (2007). Accurate quasiparticle spectra from self-consistent *GW* calculations with vertex corrections. *Phys. Rev. Lett.* **99**:246403. DOI:10.1103/PhysRevLett.99.246403
54. Albrecht, S., Reining, L., Del Sole, R. et al. (1998). Ab initio calculation of excitonic effects in the optical spectra of semiconductors. *Phys. Rev. Lett.* **80**:4510–4513. DOI:10.1103/PhysRevLett.80.4510
55. Rohlfing, M. and Louie, S.G. (1998). Electron-hole excitations in semiconductors and insulators. *Phys. Rev. Lett.* **81**:2312–2315. DOI:10.1103/PhysRevLett.81.2312
56. Li, X., Wu, X., Li, Z. et al. (2012). Bipolar magnetic semiconductors: a new class of spintronics materials. *Nanoscale* **4**:5680–5685. DOI:10.1039/C2NR31743E

57. Li, X. and Yang, J. (2016). First-principles design of spintronics materials. *Natl. Sci. Rev.* **3**:365–381. DOI:10.1093/nsr/nww026
58. Li, J., Li, X. and Yang, J. (2022). A review of bipolar magnetic semiconductors from theoretical aspects. *Fundam. Res.* **2**:511–521. DOI:10.1016/j.fmre.2022.04.002
59. Jiang, Z., Liu, Z., Li, Y. et al. (2017). Scaling universality between band gap and exciton binding energy of two-dimensional semiconductors. *Phys. Rev. Lett.* **118**:266401. DOI:10.1103/PhysRevLett.118.266401
60. Cudazzo, P., Tokatly, I.V. and Rubio, A. (2011). Dielectric screening in two-dimensional insulators: Implications for excitonic and impurity states in graphane. *Phys. Rev. B* **84**:085406. DOI:10.1103/PhysRevB.84.085406
61. Gajdoš, M., Hummer, K., Kresse, G. et al. (2006). Linear optical properties in the projector-augmented wave methodology. *Phys. Rev. B* **73**:045112. DOI:10.1103/PhysRevB.73.045112
62. Zhang, S., Sheng, H., Song, Z. et al. (2023). VASP2KP: k ·p models and Landé g-factors from ab initio calculations. *Chin. Phys. Lett.* **40**:127101. DOI:10.1088/0256-307X/40/12/127101
63. Cudazzo, P., Sponza, L., Giorgetti, C. et al. (2016). Exciton band structure in two-dimensional materials. *Phys. Rev. Lett.* **116**:066803. DOI:10.1103/PhysRevLett.116.066803
64. Egerton, R. (2011). Electron Energy-Loss Spectroscopy in the Electron Microscope (New York: Springer)
65. Schülke, W. (2007). Electron Dynamics by Inelastic X-Ray Scattering (Oxford: Oxford University Press)
66. Ament, L.J.P., van Veenendaal, M., Devereaux, T.P. et al. (2011). Resonant inelastic x-ray scattering studies of elementary excitations. *Rev. Mod. Phys.* **83**:705–767. DOI:10.1103/RevModPhys.83.705
67. He, W., Sears, J., Barantani, F. et al. (2025). Dispersive dark excitons in van der Waals ferromagnet CrI_3. *Phys. Rev. X* **15**:011042. DOI:10.1103/PhysRevX.15.011042
68. Sethi, G., Cuma, M. and Liu, F. (2023). Excitonic condensate in flat valence and conduction bands of opposite chirality. *Phys. Rev. Lett.* **130**:186401. DOI:10.1103/PhysRevLett.130.186401
69. Jan, J.F. and Lee, Y.C. (1998). Bose-Einstein condensation of excitons in two dimensions. *Phys. Rev. B* **58**:R1714. DOI:10.1103/PhysRevB.58.R1714
70. Liu, J., Qu, H. and Li, Y. (2024). One-dimensional magnetic excitonic insulators. *New J. Phys.* **26**:103034. DOI:10.1088/1367-2630/ad8955
71. Qu, H., Zhang, Z. and Li, Y. (2025). Identifying topological excitonic insulators via bulk-edge correspondence. *Phys. Rev. B* **112**:115126. DOI:10.1103/9zkm-4hmf

The Innovation, Volume 7

Supplemental Information

Spin-polarized triplet excitonic insulators in Ta_3X_8 (X = I or Br) monolayers

Haohao Sheng, Jingyu Yao, Sheng Zhang, Quansheng Wu, Zhong Fang, Xi Dai, Hongming Weng, and Zhijun Wang

A. Calculation and Methodology

We carried out first-principles calculations based on density functional theory (DFT) with the projector augmented wave (PAW) method,[1,2] as implemented in the Vienna *ab initio* simulation package (VASP).[3,4] The generalized gradient approximation (GGA) in the form of the Perdew-Burke-Ernzerhof (PBE) functional[5] was employed for the exchange-correlation potential. The kinetic energy cutoff for the plane-wave expansion was set to 330 eV. The Brillouin zone was sampled by the Monkhorst-Pack method in the self-consistent process, with a 6×6×1 **k**-mesh. The thickness of the vacuum layer along c (z) direction was set to > 15 Å. Both lattice parameters and atomic positions were fully relaxed by minimizing the interionic forces below 10^{-3} eV/Å. Spin–orbit coupling (SOC) was included in the electronic structure calculations. The PBE+U method[6–9] and the Heyd–Scuseria–Ernzerhof (HSE06) hybrid functional[10] were employed to check the electronic structure. Based on a previous report for the ferromagnetic (FM) Ta_3I_8 monolayer,[11] we set U at 2 eV. Phonon spectra were obtained using the finite-difference method with a 3×3×1 supercell, as implemented in the Phonopy package.[12] *Ab initio* molecular dynamics (AIMD) simulations were performed using the Nose–Hoover thermostat for 5000 fs with a time step of 1 fs, and the temperature was maintained at 300 K in a 3×3×1 supercell.[13] The generalized momentum matrix element $\boldsymbol{\pi}_{cv}(\boldsymbol{k})$ was calculated using the `vmat=11` function in the VASP2KP package.[14]

In order to consider many-body effects and obtain a more accurate band structure, we carried out the full-frequency single-shot GW calculations (G_0W_0)[15–18] at the PBE level, as implemented in VASP. We also performed partially self-consistent GW calculations ($EVGW_0$) by only iterating G three times to validate the results. In addition, the G_0W_0 calculations at different levels, including PBE+U and HSE06, were performed to further check the results. The GW calculations at the PBE, PBE+U, and HSE06 levels have all demonstrated high reliability in the investigation of excited states.[19–26] Quasi-particle corrections in GW calculations were both **k**-point and band dependent. We employed GW pseudopotentials, which are specifically optimized for accurately treating unoccupied states far above the Fermi level. The same **k**-mesh and a total of 400 bands (305 empty bands) were used. The energy cutoffs for the plane-wave basis set and the response function were 330 eV and 220 eV, respectively, and the soft cutoff for the Coulomb kernel in the response function was 176 eV. The number of frequency grid points was set to 100. After the GW calculations, the band structure was obtained via Wannier interpolation. The maximally localized Wannier functions for the Ta d and I/Br p orbitals were constructed using the Wannier90 package.[27,28]

In order to analyze excitonic properties, we solved the Bethe-Salpeter equation (BSE) under the Tamm-Dancoff approximation using the exact diagonalization algorithm,[29,30] as implemented in VASP. The BSE calculations were performed on top of the GW electronic structure (GW+BSE). Ten valence bands and ten conduction bands were included to build the BSE Hamiltonian. The SOC effect was included in all GW+BSE calculations.

B. Synthesizability and stability

In this section, we carefully consider the synthesizability and stability of Ta_3X_8 (X=I, Br) FM monolayers. First, we perform the convex hull analysis of formation energies for the Ta-I and Ta-Br binary systems. As shown in Figures S1A & D, the energies above the convex hull (E_{hull}) are only 0.01 eV/atom and 0.06 eV/atom for Ta_3I_8 and Ta_3Br_8, respectively. The materials with E_{hull} lower than 0.10 eV/atom are commonly regarded as experimentally accessible candidates.[31,32] Therefore, from this perspective, Ta_3X_8 FM monolayers can be synthesized experimentally. Next, we examine the dynamical and thermal stability via phonon spectra calculations and AIMD simulations. The absence of imaginary frequencies in the phonon dispersion, depicted in Figures S1B & E, confirms that Ta_3I_8 and Ta_3Br_8 FM monolayers are dynamically stable. The AIMD simulations of Ta_3I_8 and Ta_3Br_8 in Figures S1C & F reveal that at 300 K, the free energy fluctuates around a certain value without significant energy decrease over a wide range. Additionally, throughout the 5000 fs simulation, the structure remains intact, indicating thermal stability at room temperature.

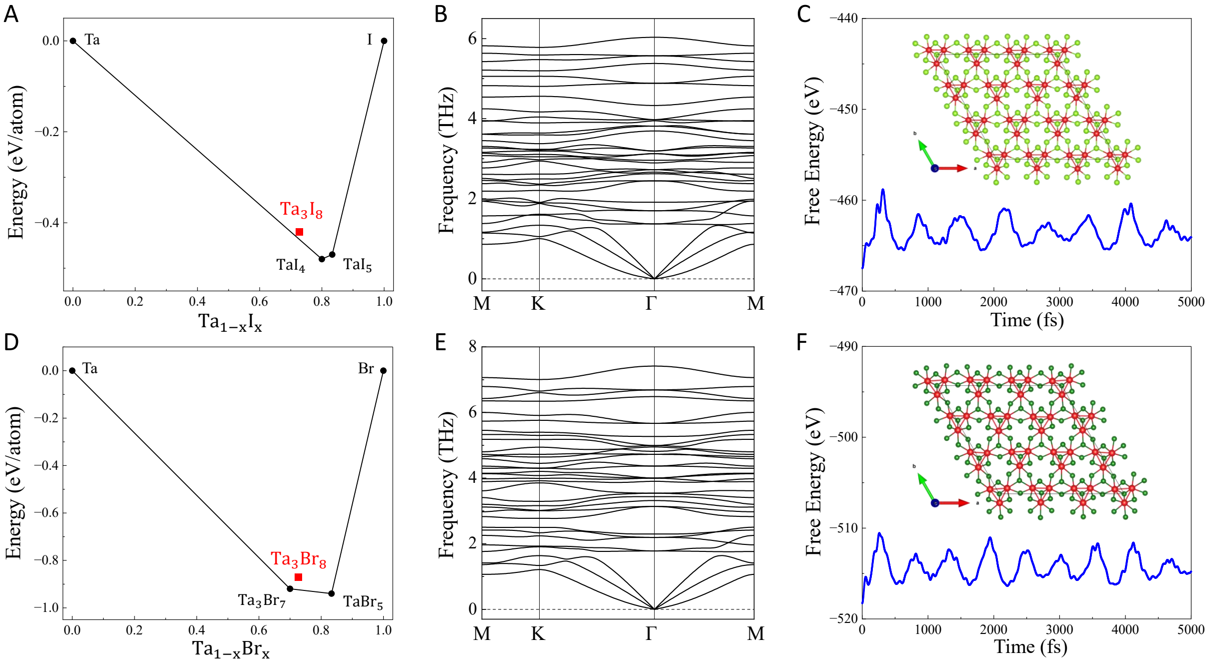

Figure S1. (A, D) Convex hull diagram for the (A) Ta-I and (D) Ta-Br binary systems. The data are sourced from Computational 2D Materials Database.[33,34] (B, E) Phonon spectra of (B) Ta_3I_8 and (E) Ta_3Br_8 FM monolayers. (C, F) AIMD simulations at 300 K of (C) Ta_3I_8 and (F) Ta_3Br_8 FM monolayers. The free energy as a function of simulation time and the structure after the 5000 fs simulation are shown.

C. Convergence tests of GW+BSE calculations at the PBE level

In this section, we thoroughly examine the convergence of GW+BSE calculations at the PBE level. Taking the FM Ta_3I_8 monolayer as an example, we carry out convergence tests of G_0W_0+BSE calculations at the PBE level using a range of different precision parameters, including **k**-mesh, the energy cutoff for the plane-wave basis set (ENCUT), and the total number of bands (NBANDS). When changing ENCUT, the energy cutoff for the response function (ENCUTGW) is set to 2/3 of ENCUT, and the soft cutoff for the Coulomb kernel in the response function (ENCUTGWSOFT) is set to 4/5 of ENCUTGW. The results for the indirect and direct single-particle band gaps (E_g), along with the binding energy (E_b) of the lowest-energy direct exciton, are shown in Figures S2A-C. These results obtained with higher precision parameters show very little variation compared to those presented in the main text, confirming that our G_0W_0+BSE calculations are well converged with respect to these parameters. In addition, we perform partially self-consistent GW calculations ($EVGW_0$) by only iterating G three times. The obtained E_b (1.450 eV) remains larger than E_g (1.393 eV). Therefore, our GW+BSE calculations at the PBE level are highly reliable.

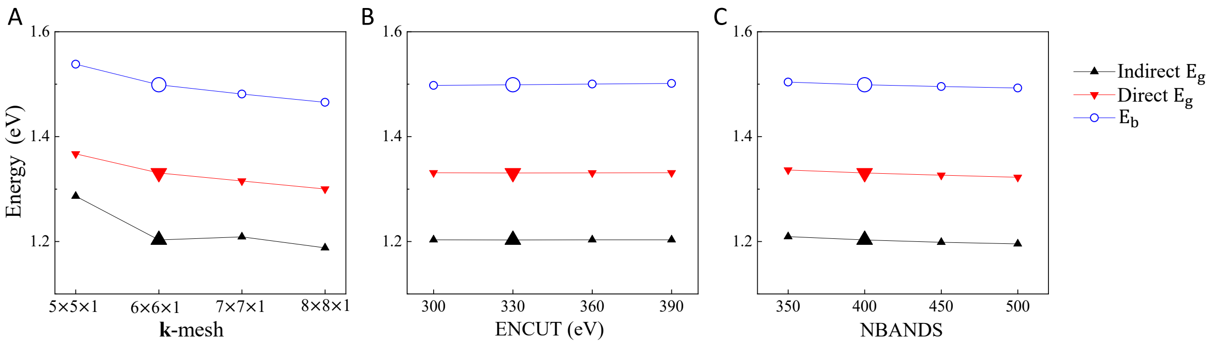

Figure S2. Convergence tests of G_0W_0+BSE calculations at the PBE level for Ta_3I_8 FM monolayer. The results, including the indirect and direct single-particle gaps (E_g) and the exciton binding energy (E_b) of the lowest-energy direct exciton, are shown as functions of (A) **k**-mesh, (B) the energy cutoff for the plane-wave basis set (ENCUT), and (C) the total number of bands (NBANDS). The enlarged data points correspond to the results used in the main text.

D. G_0W_0+BSE calculations at different levels

In this section, we provide a detailed examination of G_0W_0+BSE calculations at different levels, including PBE+U and HSE06. We take the FM Ta_3I_8 monolayer as an example. Figure S3 presents the band structures obtained using the PBE+U and HSE06 methods, as well as the G_0W_0-corrected band structures based on the respective ground states. For each method, the G_0W_0 correction primarily increases E_g without significantly altering the overall band structure. The G_0W_0 band structures at two levels are similar, with the only significant difference being an increase in E_g. Figures S3D & H show the exciton transition energies (E_t) for all direct excitons, obtained from G_0W_0+BSE calculations at the PBE+U and HSE06 levels, respectively. The E_g and the E_b of the lowest-energy exciton from different methods are presented in Figure S4. These results indicate that the E_b consistently exceeds the G_0W_0 E_g for each method. Therefore, the conclusion that the Ta_3I_8 monolayer belongs to a spin-polarized triplet excitonic insulator (EI) state is strongly supported.

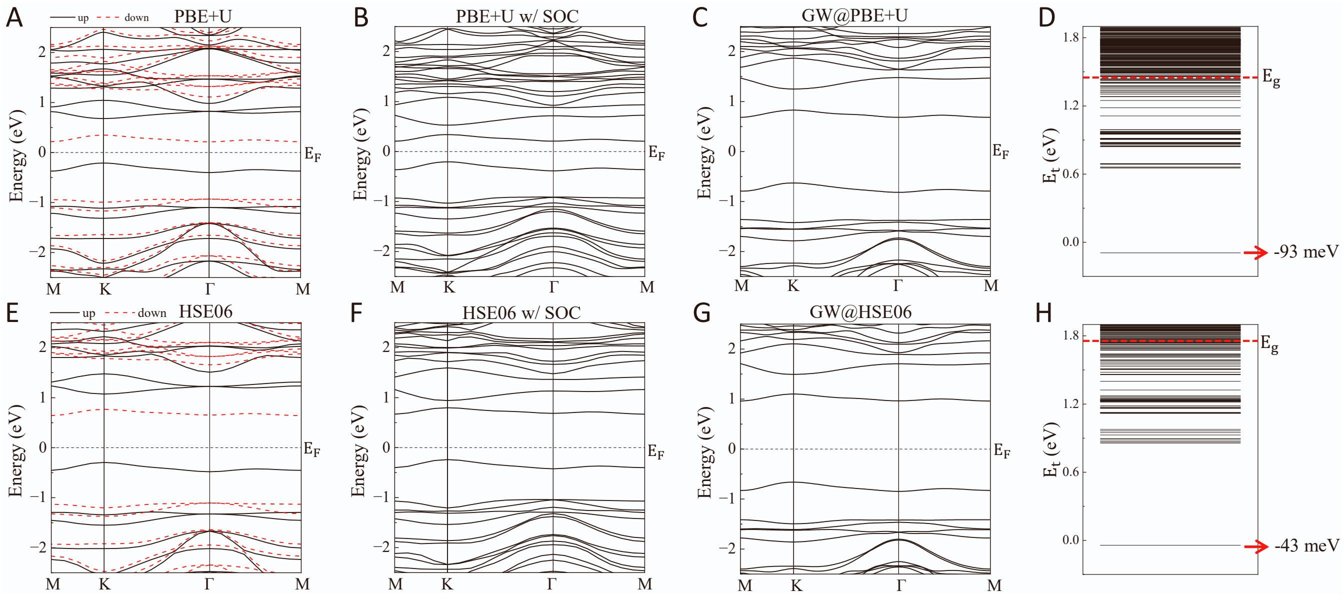

Figure S3. Band structures of Ta_3I_8 FM monolayer calculated using the (A, B) PBE+U and (E, F) HSE06 methods, both without and with SOC. Panels (C) and (G) present the G_0W_0 band structures with SOC at the PBE+U and HSE06 levels, respectively. Exciton transition energy (E_t) spectrum of direct excitons from G_0W_0+BSE calculations at the (D) PBE+U and (H) HSE06 levels. Each horizontal line corresponds to an exciton state. The lowest-energy exciton exhibits a negative E_t.

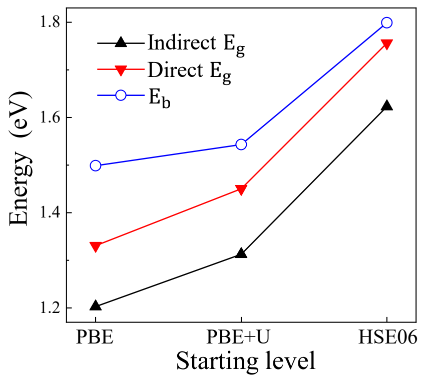

Figure S4. G_0W_0+BSE calculations at the PBE, PBE+U, and HSE06 levels for Ta_3I_8 FM monolayer. The E_g and the E_b of the lowest-energy direct exciton are shown. The E_b consistently exceeds the E_g at each method.

E. External magnetic field, electric field, and strain

In this section, we investigate the influences of external magnetic field, electric field, and strain on the electronic structure for Ta_3I_8 FM monolayer. First, we analyze the influence of an external magnetic field. For this purpose, we calculate the magnetic anisotropy energy. The total energy as a function of magnetization direction is shown in Figure S5. Ta_3I_8 displays out-of-plane (xz-plane) anisotropy, with the energy of the x-directed FM configuration being 0.358 meV per Ta atom lower than that of the z-directed FM configuration. However, the energy differences for the in-plane (xy-plane) directions are less than 0.001 meV. These results indicate that the in-plane FM configuration is the most favorable, and the moment orientation can be effectively tuned by an external magnetic field. We perform G_0W_0+BSE calculations at the PBE level for three FM configurations, where the magnetic moments are aligned parallel to the x, y, and z directions. For these FM configurations, two low-energy flat bands that determine the formation of EI are almost identical, as confirmed by the G_0W_0 band structures in Figure S6. Further BSE calculations show that the E_t values of the lowest-energy direct exciton are -167 meV, -167 meV, and -168 meV, respectively. Therefore, the influence of magnetic direction on the spin-polarized triplet EI state is negligible.

Second, we investigate the effects of electric field and strain on the electronic structure. The spin-polarized band structures under ± 0.5 V/Å vertical electric fields and $\pm 3\%$ biaxial strains are shown in Figures S7B-E. The biaxial strain is defined as $\epsilon = \frac{a-a_0}{a_0} \times 100\%$, where a and a_0 are the lattice constants in the condition of strain and equilibrium. We can observe that, under such electric fields and strains, the two low-energy bands that determine the formation of EI have not undergone any significant changes. Therefore, we conclude that the spin-polarized triplet EI state remains robust under a weak electric field and strain.

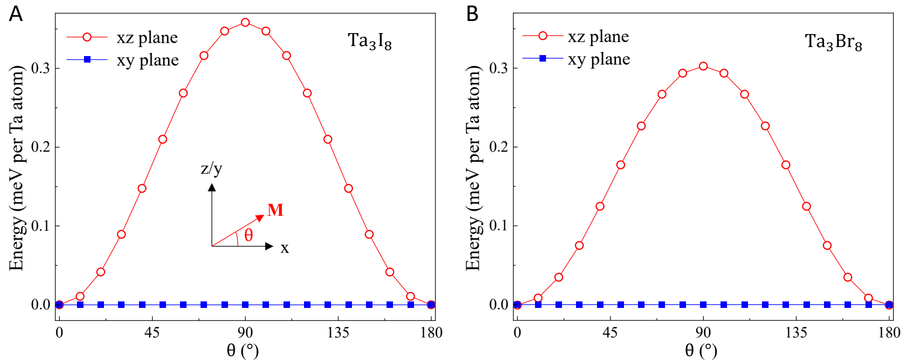

Figure S5. The total energies as a function of magnetization direction in xz and xy planes for (A) Ta_3I_8 and (B) Ta_3Br_8 FM monolayers.

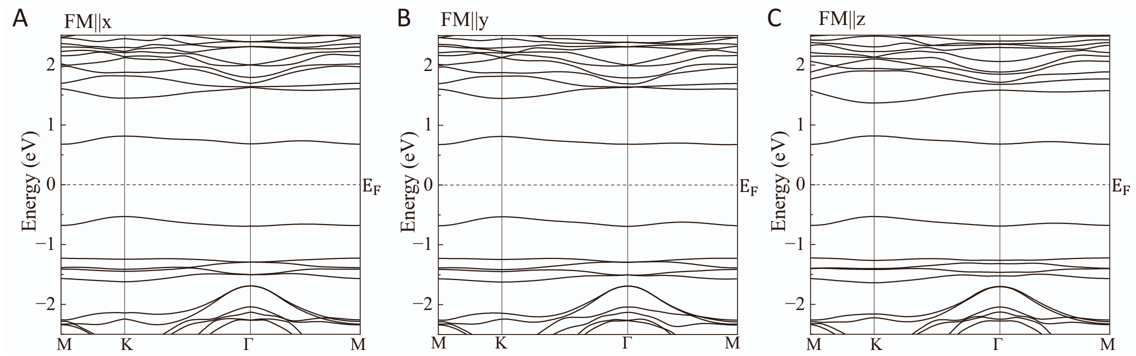

Figure S6. The band structures with SOC from G_0W_0 calculations at the PBE level for Ta_3I_8 FM monolayer, where the magnetic moments are aligned parallel to the (A) x, (B) y, and (C) z directions.

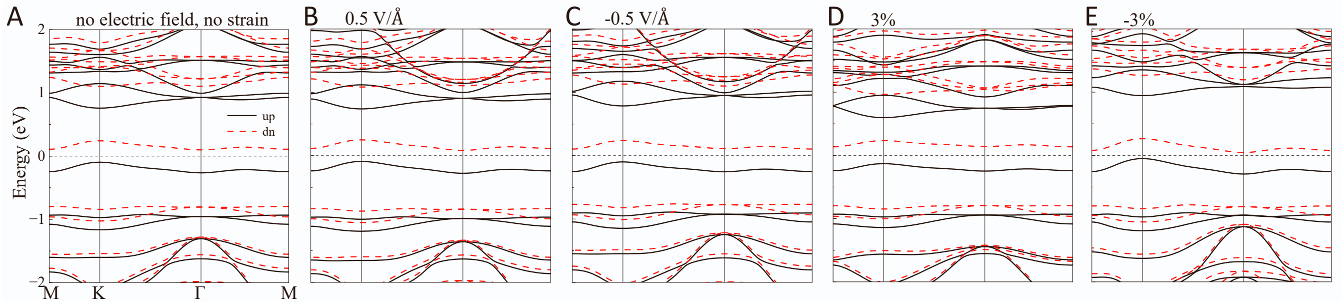

Figure S7. Spin-polarized band structures of Ta_3I_8 FM monolayer, under (A) no vertical electric field and no biaxial strain, (B) 0.5 V/Å vertical electric field, (C) -0.5 V/Å vertical electric field, (D) 3% biaxial strain, and (E) -3% biaxial strain.

F. Electronic structures and low-energy excitons of Ta_3Br_8 monolayer

In this section, we analyze the electronic structures and low-energy excitons of FM Ta_3Br_8 monolayer. Figure S8A presents the spin-polarized band structure and partial densities of states without SOC. The highest valence band (VB) and the lowest conduction band (CB) exhibit opposite spin directions and show minimal energy dispersion. The two low-energy flat bands primarily originate from Ta d_{z^2} orbitals. SOC has little impact on the low-energy flat bands. The same-orbital parity and opposite-spin nature of the band-edge states effectively suppress dielectric screening, promoting the emergence of the EI state.

To analyze low-energy excitons, we perform GW+BSE calculations at the PBE level. As shown in Figure S8B, a more accurate band structure is obtained by many-body single-shot GW calculations (G_0W_0) at the PBE level, where the E_g changes from 0.386 eV (PBE) to 1.722 eV (G_0W_0). Based on the G_0W_0 electronic structure, we solve the BSE using ten VBs and ten CBs. As shown in Figure S8C, the lowest-energy dark direct exciton hosts a negative E_t = -264 meV, indicating that the E_b (1.986 eV) exceeds the E_g. This implies that the Ta_3Br_8 FM monolayer exhibits a many-body EI ground state. We further find that the lowest-energy exciton arises solely from the highest VB and the lowest CB. The spin-flipping in spin-polarized electron-hole transitions can produce spin-polarized triplet excitons with a finite spin moment. Therefore, FM Ta_3Br_8 monolayer is a spin-polarized triplet EI, where the spontaneous BEC of excitons can generate an intriguing spin supercurrent.

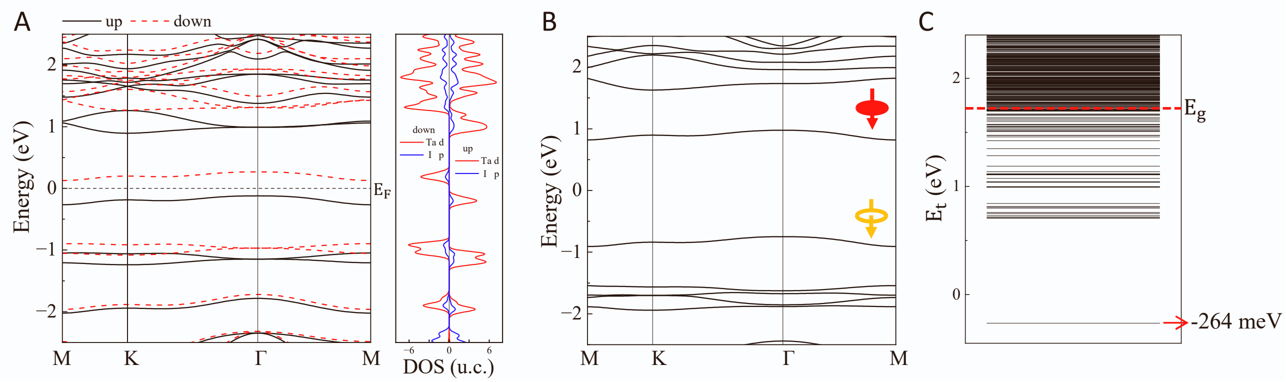

Figure S8. Electronic structure and low-energy excitons of Ta_3Br_8 FM monolayer. (A) The spin-polarized band structure and partial densities of states (DOS) without SOC. (B) The band structure with SOC from G_0W_0 calculations at the PBE level. For the lowest-energy exciton generated by the transition between the two low-energy bands, the orange circled arrow indicates the spin of the hole remaining after excitation, while the red arrow denotes the spin of the excited electron. (C) Exciton transition energy (E_t) spectrum. Each horizontal line corresponds to an exciton state. The lowest-energy exciton exhibits a negative E_t.

REFERENCES

[1] Blöchl P.E. (1994). Projector augmented-wave method. *Phys. Rev. B* **50**:17953. DOI:10.1103/PhysRevB.50.17953

[2] Kresse G. and Joubert D. (1999). From ultrasoft pseudopotentials to the projector augmented-wave method. *Phys. Rev. B* **59**:1758. DOI:10.1103/PhysRevB.59.1758

[3] Kresse G. and Furthmüller J. (1996). Efficiency of ab-initio total energy calculations for metals and semiconductors using a plane-wave basis set. *Comput. Mater. Sci.* **6**:15. DOI:10.1016/0927-0256(96)00008-0

[4] Kresse G. and Furthmüller J. (1996). Efficient iterative schemes for *ab initio* total-energy calculations using a plane-wave basis set. *Phys. Rev. B* **54**:11169. DOI:10.1103/PhysRevB.54.11169

[5] Perdew J.P., Burke K. and Ernzerhof M. (1996). Generalized gradient approximation made simple. *Phys. Rev. Lett.* **77**:3865. DOI:10.1103/PhysRevLett.77.3865

[6] Anisimov V.I., Zaanen J. and Andersen O.K. (1991). Band theory and Mott insulators: Hubbard U instead of Stoner I. *Phys. Rev. B* **44**:943. DOI:10.1103/PhysRevB.44.943

[7] Anisimov V.I., Solovyev I.V., Korotin M.A. *et al.* (1993). Density-functional theory and NiO photoemission spectra. *Phys. Rev. B* **48**:16929. DOI:10.1103/PhysRevB.48.16929

[8] Dudarev S.L., Botton G.A., Savrasov S.Y. *et al.* (1998). Electron-energy-loss spectra and the structural stability of nickel oxide: An LSDA+U study. *Phys. Rev. B* **57**:1505. DOI:10.1103/PhysRevB.57.1505

[9] Anisimov V.I., Aryasetiawan F. and Lichtenstein A.I. (1997). First-principles calculations of the electronic structure and spectra of strongly correlated systems: the LDA+U method. *J. Phys.: Condens. Matter* **9**:767. DOI:10.1088/0953-8984/9/4/002

[10] Krukau A.V., Vydrov O.A., Izmaylov A.F. *et al.* (2006). Influence of the exchange screening parameter on the performance of screened hybrid functionals. *J. Chem. Phys.* **125**:224106. DOI:10.1063/1.2404663

[11] Xing S., Wang B., Zhao T. *et al.* (2024). Independent electrical control of spin and valley degrees in 2D breathing kagome Ta_3I_8 with intrinsic triferroicity. *J. Phys. Chem. Lett.* **15**:6489. DOI:10.1021/acs.jpclett.4c00858

[12] Togo A. and Tanaka I. (2015). First principles phonon calculations in materials science. *Scr. Mater.* **108**:1. DOI:10.1016/j.scriptamat.2015.07.021

[13] Nosé S. (1984). A unified formulation of the constant temperature molecular dynamics methods. *J. Chem. Phys.* **81**:511. DOI:10.1063/1.447334

[14] Zhang S., Sheng H., Song Z. *et al.* (2023). VASP2KP: $k\cdot p$ models and Landé g-factors from *ab initio* calculations. *Chin. Phys. Lett.* **40**:127101. DOI:10.1088/0256-307X/40/12/127101

[15] Shishkin M. and Kresse G. (2006). Implementation and performance of the frequency-dependent GW method within the PAW framework. *Phys. Rev. B* **74**:035101. DOI:10.1103/PhysRevB.74.035101

[16] Shishkin M. and Kresse G. (2007). Self-consistent GW calculations for semiconductors and insulators. *Phys. Rev. B* **75**:235102. DOI:10.1103/PhysRevB.75.235102

[17] Fuchs F., Furthmüller J., Bechstedt F. *et al.* (2007). Quasiparticle band structure based on a generalized Kohn-Sham scheme. *Phys. Rev. B* **76**:115109. DOI:10.1103/PhysRevB.76.115109

[18] Shishkin M., Marsman M. and Kresse G. (2007). Accurate quasiparticle spectra from self-consistent GW calculations with vertex corrections. *Phys. Rev. Lett.* **99**:246403. DOI:10.1103/PhysRevLett.99.246403

[19] Jiang Z., Liu Z., Li Y. *et al.* (2017). Scaling universality between band gap and exciton binding energy of two-dimensional semiconductors. *Phys. Rev. Lett.* **118**:266401. DOI:10.1103/PhysRevLett.118.266401

[20] Jiang Z., Li Y., Duan W. *et al.* (2019). Half-excitonic insulator: A single-spin Bose-Einstein condensate. *Phys. Rev. Lett.*

122:236402. DOI:10.1103/PhysRevLett.122.236402

[21] Jiang Z., Lou W., Liu Y. *et al.* (2020). Spin-triplet excitonic insulator: The case of semihydrogenated graphene. *Phys. Rev. Lett.* **124**:166401. DOI:10.1103/PhysRevLett.124.166401

[22] Yao J., Sheng H., Zhang R. *et al.* (2024). Excitonic instability in $Ta_2Pd_3Te_5$ monolayer. *Chin. Phys. Lett.* **41**:097101. DOI:10.1088/0256-307X/41/9/097101

[23] Kharche N., Muckerman J.T. and Hybertsen M.S. (2014). First-principles approach to calculating energy level alignment at aqueous semiconductor interfaces. *Phys. Rev. Lett.* **113**:176802. DOI:10.1103/PhysRevLett.113.176802

[24] Karlický F. and Otyepka M. (2013). Band gaps and optical spectra of chlorographene, fluorographene and graphane from G_0W_0, GW_0 and GW calculations on top of PBE and HSE06 orbitals. *J. Chem. Theory Comput.* **9**:4155. DOI:10.1021/ct400476r

[25] Rodrigues Pela R., Vona C., Lubeck S. *et al.* (2024). Critical assessment of G_0W_0 calculations for 2D materials: the example of monolayer MoS_2. *npj Comput. Mater.* **10**:77. DOI:10.1038/s41524-024-01253-2

[26] Dong S., Chen Y., Qu H. *et al.* (2025). Topological exciton density wave in monolayer WSe_2. *Phys. Rev. Lett.* **134**:066602. DOI:10.1103/PhysRevLett.134.066602

[27] Mostofi A.A., Yates J.R., Pizzi G. *et al.* (2014). An updated version of wannier90: A tool for obtaining maximally-localised Wannier functions. *Comput. Phys. Commun.* **185**:2309. DOI:10.1016/j.cpc.2014.05.003

[28] Marzari N., Mostofi A.A., Yates J.R. *et al.* (2012). Maximally localized Wannier functions: Theory and applications. *Rev. Mod. Phys.* **84**:1419. DOI:10.1103/RevModPhys.84.1419

[29] Albrecht S., Reining L., Del Sole R. *et al.* (1998). *Ab initio* calculation of excitonic effects in the optical spectra of semiconductors. *Phys. Rev. Lett.* **80**:4510. DOI:10.1103/PhysRevLett.80.4510

[30] Rohlfing M. and Louie S.G. (1998). Electron-hole excitations in semiconductors and insulators. *Phys. Rev. Lett.* **81**:2312. DOI:10.1103/PhysRevLett.81.2312

[31] Zeni C., Pinsler R., Zügner D. *et al.* (2025). A generative model for inorganic materials design. *Nature* **639**:624. DOI:10.1038/s41586-025-08628-5

[32] Park H., Onwuli A. and Walsh A. (2025). Exploration of crystal chemical space using text-guided generative artificial intelligence. *Nat. Commun.* **16**:4379. DOI:10.1038/s41467-025-59636-y

[33] Haastrup S., Strange M., Pandey M. *et al.* (2018). The computational 2D materials database: high-throughput modeling and discovery of atomically thin crystals. *2D Mater.* **5**:042002. DOI:10.1088/2053-1583/aacfc1

[34] Gjerding M.N., Taghizadeh A., Rasmussen A. *et al.* (2021). Recent progress of the Computational 2D Materials Database (C2DB). *2D Mater.* **8**:044002. DOI:10.1088/2053-1583/ac1059
